# Endothelial oncogenic KRAS mutation drives the dynamics of microglia and macrophages in brain arteriovenous malformation

**DOI:** 10.1172/jci.insight.195638

**Published:** 2026-02-05

**Authors:** Hyejin Park, Jung-Eun Park, Bridger H. Freeman, Bosco Seong Kyu Yang, Shun-Ming Ting, Alexander K. Suh, Jude P.J. Savarraj, Shuning Huang, Jakob Körbelin, Huimahn Alex Choi, Sean P. Marrelli, Jaroslaw Aronowski, Peng Roc Chen, Eunhee Kim, Eun S. Park

**Affiliations:** 1 Vivian L. Smith Department of Neurosurgery,; 2 Department of Neurology, and; 3 Department of Diagnostic and Interventional Imaging, McGovern Medical School, The University of Texas Health Science Center at Houston, Houston, Texas, USA.; 4 Department of Oncology, Hematology and Bone Marrow Transplantation, University Medical Center Hamburg-Eppendorf, Hamburg, Germany.; 5 Center for Neuroimmunology and Glial Biology, The Brown Foundation Institute of Molecular Medicine, The University of Texas Health Science Center at Houston, Houston, Texas, USA.

**Keywords:** Neuroscience, Vascular biology, Endothelial cells, Macrophages, Stroke

## Abstract

Mutation of KRAS in endothelial cells (KRAS-ECs) leads to intracerebral hemorrhage (ICH) in brain arteriovenous malformation (bAVM), resulting in severe disabilities or even death. However, it is unclear what causes this hemorrhagic conversion of bAVMs. Here, using a locally established, clinically relevant sporadic bAVM mouse model, created by overexpressing mutant KRAS (KRAS^G12V^) in brain ECs, we demonstrate that KRAS-ECs act as trigger for activation of microglia (MG) and infiltration of macrophages (Mϕ). Using a 3-dimensional immunostaining approach with cleared human and mouse bAVM tissues, we demonstrate an abundance of MG/Mϕ around the bAVM nidus. The presence of MG/Mϕ was correlated to the blood-brain barrier leakage in bAVM areas. Time-lapsed intravital imaging in *Cx3cr1-gfp;Ccr2-rfp* reporter mice demonstrated the dynamic activation of MG and infiltration of Mϕ toward mutant KRAS^G12V^–modified dysplastic vessels. Importantly, a time-course analysis showed that these activated MG and infiltrated Mϕ are present around the bAVMs prior to hemorrhagic conversion, and controlled depletion of MG/Mϕ reduced ICH incidence in bAVMs. Inhibition of MG/Mϕ with long-term minocycline treatment attenuated the incidence of ICHs around bAVMs. Our study indicates that MG/Mϕ are involved in destabilization of KRAS^G12V^-induced bAVM, leading to hemorrhagic conversion/ICH. Thus, modulation of MG/Mϕ may reduce ICH risk in patients with bAVM.

## Abstract 2

The study focuses on the mechanism of intracerebral hemorrhage by understanding the role of microglia and macrophages in mutant KRAS-induced brain arteriovenous malformation.

## Graphical abstract

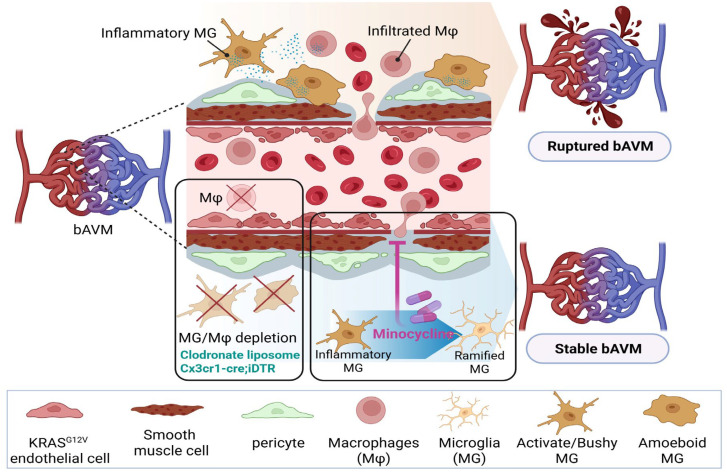

## Introduction

Brain arteriovenous malformations (bAVMs) are tangled blood vessels in the brain, formed by direct connection between arteries and veins without an intervening microcapillary system (1). A bAVM can easily rupture, causing intracerebral hemorrhage (ICH), which leads to 40%–70% of deaths in patients with bAVM (2–4). Moreover, bAVM-associated bleeding is the most common cause of hemorrhagic stroke in children (5). A surgical resection of aberrant vessels is often performed to treat bAVMs. However, there are significant side effects and risks associated with invasive surgery, particularly when the bAVM is located in an eloquent region of the brain. In patients younger than 25 years old, there is a 5% risk of bAVM recurrence, even after the initial surgical obliteration (1, 6). Thus, there is an urgent need to understand the inciting pathophysiology and develop targeted pharmacological therapies for patients with bAVM.

Most bAVMs are typically sporadic, with only 5% of bAVMs associated with familial mutations, such as the hereditary hemorrhagic telangiectasia (HHT) genes, *ENG* or *ALK1*, which encode proteins that mediate TGF-β family coreceptor signaling, distorting vessel morphology and increasing the risk for ICH (7–10). Recent studies revealed that somatic activating KRAS mutations (including Gly12Val and Gly12Asp) were detected specifically in brain endothelial cells (ECs) from 72% of patients with sporadic bAVM with an ICH (11–13). In preclinical studies, the capacity of endothelial KRAS mutations to trigger bAVM genesis was demonstrated in mice (14–16). These studies showed that the brain EC–specific adeno-associated virus (AAV) capsid (AAV-BR1) delivering mutated KRAS was sufficient to trigger formation of dysplastic vessels, effectively modeling bAVMs in mice. Our locally established bAVM mouse model that specifically transduces brain vascular ECs (bECs) (KRAS^G12V/bEC^ mice) using AAV-BR1-KRAS^G12V^ successfully recapitulated the human bAVM pathology, including tangled/snarled vasculature, spontaneous ICH, and neurological deficits. In KRAS^G12V/bEC^ mice, the spontaneous extravasation of red blood cells (RBCs) was from the malformed vessels of the bAVM nidus (15). Although evidence shows that mutant KRAS is sufficient to induce bAVM pathogenesis, mechanisms leading to hemorrhagic conversion of the KRAS-associated bAVM are not clear.

Since increased myeloid cell surveillance has been observed in the hemorrhagic regions of human bAVMs and mouse models of HHT, it has been proposed that the hemorrhagic conversion of bAVMs may lead to robust activation and/or recruitment of immune cells to bleeding bAVMs (17–19). Genome-wide RNA sequencing (RNA-seq) or gene expression profiling of human bAVM tissues/blood revealed the presence of inflammatory cytokines and chemokines (20–23), which were confined primarily to microglia (MG) and macrophages (Mϕ) (together, MG/Mϕ) (24, 25). Notably, inflammation is known to impair the integrity of EC junctions and the blood-brain barrier (BBB) (26, 27), suggesting that MG/Mϕ-mediated inflammation is causally associated with vascular instability. In KRAS^G12V/bEC^ mice, we observed the robust presence of activated MG and infiltrating Mϕ at bAVM locations, which was defined by the histological area demonstrating malformed dysplastic vessels compared with the intact area (15). Our observation that activated MG and infiltrated Mϕ are located around unruptured bAVMs raised interest in the possibility that myeloid cells may indeed be involved in promoting the instability of malformed vessels in the bAVM area prior to rupture. The presence of inflammatory MG/Mϕ in the bAVM area suggests that an inhibitory strategy targeting this MG/Mϕ-associated inflammation could stabilize bAVMs. Minocycline, an FDA-approved antibiotic, has demonstrated efficacy in reducing inflammation and may be a pharmacological treatment option for patients with bAVM (28). The evidence led us to test the mechanistic role of minocycline in the mutant KRAS–induced bAVM mouse model.

In this study, we demonstrate that bAVMs formed in response to KRAS mutation drives the activation of Cx3cr1^+^ MG and infiltration of Ccr2^+^ Mϕ toward the bAVM region, as established with myeloid cell–lineage reporter mice. Early MG/Mϕ depletion using clodronate liposomes (CLs) and conditional *Cx3cr1-cre/ERT2;Rosa26-iDTR* (iDTR) mice reduced ICH incidence, demonstrating their causal role in bAVM instability and ICH. Also, treatment with minocycline, a potent immune modulator, reduced MG/Mϕ activation/infiltration and bAVM-associated ICH incidence. Importantly, we showed a robust MG/Mϕ presence in the unruptured human bAVM nidus, similar to our findings in the experimental bAVM model. Overall, our results support the notion that inhibition of MG/Mϕ-mediated inflammation around bAVMs could represent a pharmacological target to enhance bAVM stability to prevent ICH.

## Results

### ECs carrying mutant KRAS lead to bAVM genesis.

We previously showed that ECs carrying mutant human KRAS (KRAS-ECs) are sufficient to generate a tangled nidus, with feeding arteries connected directly to draining veins in the brains of mice, recapitulating the features of human bAVM (15). The bAVM mouse model was established using AAV-BR1-KRAS^G12V^ that specifically transduces bECs using a unique BR1 capsid carrying KRAS^G12V^ (29). We named these KRAS^G12V/bEC^ mice (15). Using antibodies that selectively recognize mutated human KRAS^G12V^ and CD31, we confirmed that bAVM vessels are indeed formed at the locus of KRAS^G12V^ expression (Supplemental Figure 1A; supplemental material available online with this article; https://doi.org/10.1172/jci.insight.195638DS1). The region, termed the “bAVM territory,” containing KRAS^G12V^-positive dysplastic, malformed vessels, showed a striking prevalence of activated MG and infiltrated Mϕ (Iba1^+^ cells). This is in contrast with “intact,” KRAS^G12V^-negative vessels with normal nondysplastic morphology that showed only MG with ramified morphology (Supplemental Figure 1B).

**Figure 1:**
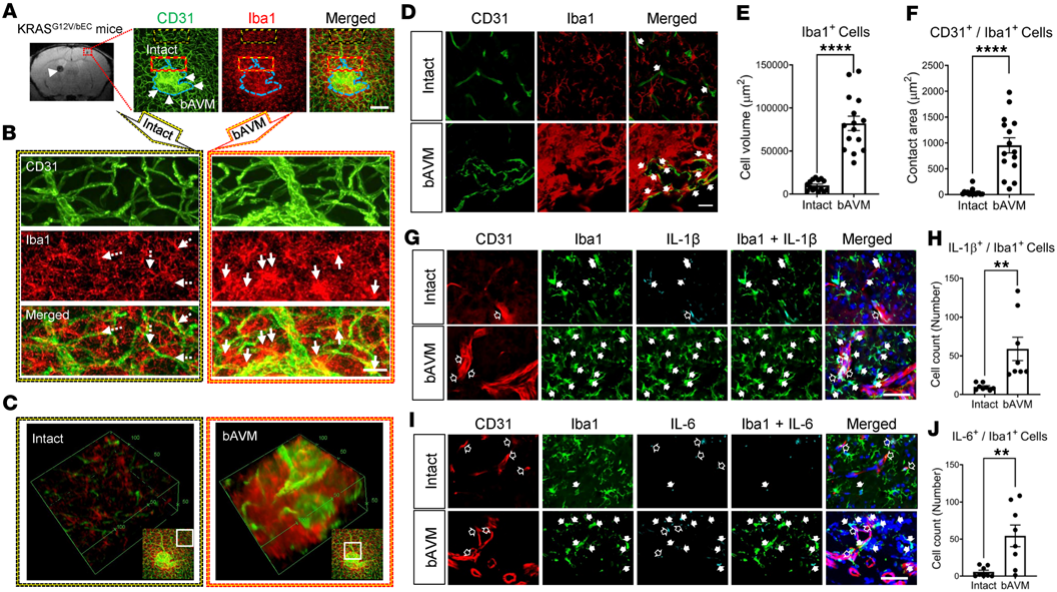
KRAS-ECs drive the activation of MG and infiltration of Mϕ toward bAVM territories in KRAS^G12V/bEC^ mice. (**A**) T_2_*-weighted MRI revealed the presence of ICH 8 weeks after AAV-BR1-KRAS^G12V^ injection. For labeling, whole brains were cleared using the tissue rapid clearing technique before being processed for immunostaining. Confocal microscopy visualized the bAVMs (tissue corresponding to the red square of the MRI image) as a dense network of CD31^+^ (EC marker) vasculature. A nidus shape of bAVM vessels (CD31, green) is distinguished from intact vessels. Dotted blue lines with arrows indicate the bAVM nidus. An abundant population of MG/Mϕ (Iba1, red) surrounding bAVMs was clearly detected. Scale bar: 100 μm. The large hypointense bleeding spot on the MRI (arrow) was not analyzed due to tissue disintegration following ICH from the bAVM nidus. (**B**) High magnification of the dotted area in **A** representing the intact brain (yellow-filled black dotted) or bAVM-containing area (yellow-filled, red dotted). Scale bar: 25 μm. Note the less dense contact between MG/Mϕ and the vasculature (CD31^+^) in the intact brain area (dotted arrows), while bAVM vessels exhibit an enhanced contact between Mg/Mϕ and bAVM vessels (arrows). (**C**) Representative 3D images reconstructing stacked images of cleared/immunofluorescently stained brain tissues. The 3D images clearly show a higher density and intensity of myeloid (Iba1^+^) cells attached to CD31^+^ dysplastic bAVM vessels, compared with the intact vessels in KRAS^G12V/bEC^ mice. Units indicate scales (149 μm × 149 μm × 67 μm). Sample thickness: 2 mm. *Z*-stacks: 67 μm. (**D**) Representative immunofluorescence images showing the increased clustering of Iba1^+^ cells in the bAVM territory compared with intact vessels 6 weeks after AAV-BR1-KRAS^G12V^ injection. Arrows indicate the contact of Iba1^+^ cells to the CD31^+^ ECs. Scale bar: 25 μm. (**E** and **F**) Bar graphs quantifying volume of Iba1^+^ cells (**E**) or contact area of Iba1^+^ cells to the CD31^+^ ECs (**F**) around intact vessels versus bAVM-containing territories. Unpaired, 2-tailed *t* test. ****P < 0.0001. Each dot indicates a randomly selected ROI (*n* = 15) obtained from mice (*n* = 6 per group). (**G** and **I**) Representative immunofluorescence images showing the enhanced production of IL-1β or IL-6 by Iba1^+^ cells in the bAVM territory compared with intact vessels 6 weeks after AAV-BR1-KRAS^G12V^ injection. Arrows indicate IL-1β^+^Iba1^+^ cells (**G**) or IL-6^+^Iba1^+^ cells (**I**). Open arrows indicate IL-1β^+^CD31^+^ cells (**G**) or IL-6^+^CD31^+^ cells (**I**). Scale bar: 50 μm. (**H** and **J**) Bar graphs quantifying numbers of IL-1β^+^Iba1^+^ (**E**) or IL-6^+^Iba1^+^ cells (**G**) around intact vessels versus bAVM-containing territories. Unpaired, 2-tailed *t* test. **P < 0.01. Each dot indicates a randomly selected ROI (*n* = 8) obtained from mice (*n* = 3 per group).

To provide better visualization of bAVM-MG/Mϕ interactions, we used a clearing technique with 3D immunofluorescent staining in the whole mouse brain 8 weeks after AAV-BR1-KRAS^G12V^ injection. Brain tissue containing the bAVMs was harvested using T_2_*-weighted MRI coordinates (Figure 1A). The cleared, CD31-immunostained brain tissues showed snarled bAVM niduses readily distinguishable from normal vessels. The 3D-stereotypic structure of bAVM niduses possessed thick and dysplastic vasculature and contained activated Iba1^+^ cells (Figure 1, A–C, and Supplemental Video 1).

### KRAS-ECs activate and recruit MG/Mϕ in the bAVM territory.

The presence of MG and monocyte-derived Mϕ in and around the tangled/dysplastic nidus suggested they potentially play a pathological role in the bAVM territory. Thus, here, to study the bAVM–myeloid cell interaction, we investigated the spatial relationship between MG/Mϕ and bAVMs, again using the whole mouse brain clearing technique with subsequent Iba1 immunofluorescent staining for MG/Mϕ. The 3D-stereotypic structure of the bAVM nidus showed dense populations of Iba1^+^ MG/Mϕ around thick and dysplastic vasculature (Figure 1, A–C, and Supplemental Video 1). Moreover, in the bAVMs, we identified that MG, exhibiting thick branches, were largely adjacent to dysplastic vessels. This is in contrast with the intact region (no-bAVM) where CD31^+^ vessels showed limited contact with ramified MG (Figure 1, B and C). The volume of Iba1^+^ cells and the contact area of Iba1^+^ cells between CD31^+^ ECs were substantially increased in the bAVM area compared with the intact area (Figure 1, D–F). As MG/Mϕ at bAVMs may influence the local proinflammatory environment, we determined at 6 weeks after bAVM induction that the number of IL-1β^+^Iba1^+^ and IL-6^+^Iba1^+^ cells was much higher around bAVMs (Figure 1, G–J). This suggests that the MG/Mϕ in bAVM territories have a proinflammatory phenotype, which could directly induce bAVM destabilization.

### Presence of MG/Mϕ in both human ruptured and unruptured bAVMs.

**Figure 2:**
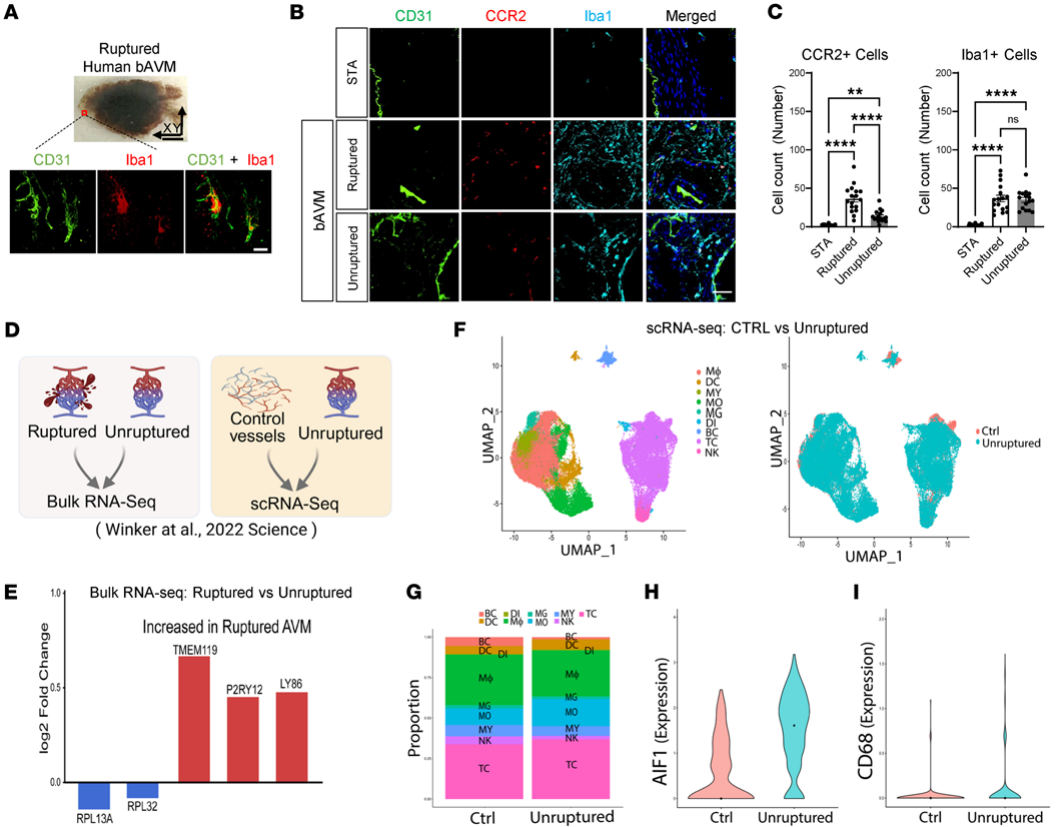
Human bAVMs display the presence of MG/Mϕ in bAVM territories. (**A**) A ruptured bAVM nidus dissected from a human patient was cleared using the tissue rapid clearing technique. bAVM nidus thickness: 2 mm. Representative immunofluorescence image of endothelium (CD31, green) and MG/Mϕ (Iba1, red) at the location (red inset of **A**) in cleared ruptured bAVM nidus. Scale bars: 200 μm (top) and 50 μm (bottom). (**B**) Representative immunofluorescence image demonstrating Iba1^+^ cells and CCR2^+^ cells in human ruptured and unruptured bAVM tissues compared to non–bAVM-associated superficial temporal artery (STA) used as control. Note that the dissected STA and bAVM are composed primarily of blood vessels and lack parenchymal tissue. Scale bar: 50 μm. (**C**) Bar graphs quantifying numbers of CCR2^+^ and Iba1^+^ cells between human STA, ruptured, and unruptured bAVM tissues. One-way ANOVA and Tukey’s multiple comparisons test. **P < 0.01; ****P < 0.0001. Each dot indicates a randomly selected ROI (*n* = 16–17) obtained from human images (*n* = 3) per group. (**D**) External validation: Reanalysis of bulk RNA-seq by comparing ruptured (*n* = 26 donors) versus unruptured human bAVM tissues (*n* = 13 donors), and scRNA-seq by comparing Ctrl (control adult vessels from epilepsy patients, *n* = 5 donors) versus unruptured human bAVM tissues (*n* = 5 donors), based on the dataset by Winkler et al. (30). Created in BioRender (https://BioRender.com/w59711f). (**E**) External validation: MG and Mϕ in the bulk RNA-seq dataset express their respective markers: *TMEM119* and *P2RY12* (MG) and *LY86* (Mϕ). Note that the MG/Mϕ genes are significantly expressed in the ruptured bAVM tissues compared with the unruptured bAVM tissues. Proportions of selected genes for MG and Mϕ were compared between ruptured and unruptured using nonparametric statistical tests (log_2_[fold change]). (**F**–**I**) External validation: UMAP visualization of immune cell types from reanalyzed scRNA-seq data of Ctrl and unruptured bAVM tissues (**F**). Mϕ, macrophages; DC, dendritic cell; MY, myeloid; MO, monocytes; MG, microglia; DI, dividing immune cells; BC, B cell; TC, T cell; NK, natural killer cells. No significant batch effect was observed. Note that the proportions of MG and Mϕ are similar compared to control vessels (**G**), while the unruptured bAVM tissues have increased *CD68* and *AIF1* in MG (**H** and **I**). All analyses were conducted in R version 4.3. Fisher’s exact tests were used to determine the statistical significance of cell proportions. The significance of selected genes was tested with Wilcoxon’s rank-sum tests to compare Ctrl and unruptured. *P* = 0.00718 (*AIF1*), *P* = 0.0241 (*CD68*).

Importantly, we confirmed consistency between mouse bAVMs and human bAVMs, now using tissue surgically resected from patients with bAVM. We again used the tissue clearing technique and immunofluorescent staining for Iba1 to achieve better resolution. The clustering of Iba1^+^ MG/Mϕ in the human ruptured bAVMs was morphologically similar to what we observed in the mouse model (Figure 2A and Supplemental Video 2). Along with this, we confirmed that ruptured bAVM tissues had substantially increased numbers of Iba1^+^ and CCR2^+^ cells compared with superficial temporal artery (STA) tissues (Figure 2, B and C, Supplemental Table 1). For testing the MG/Mϕ changes in human ruptured versus unruptured bAVM, we reanalyzed the human bAVM bulk RNA-seq dataset established by Winkler et al. (30). Notably, the MG-specific genes (transmembrane protein 119 [*TMEM119*], *P2RY12*) or perivascular Mϕ-specific genes (*LY86*) were both increased in ruptured bAVM compared with the unruptured bAVM tissues (Figure 2E). Remarkably, using the human bAVM single-cell RNA-seq (scRNA-seq) dataset (30), we reanalyzed the immune cell composition across control (vessels from patients with epilepsy) and unruptured bAVM tissues. The relative proportions of MG and Mϕ were broadly similar between groups. MG accounted for a median of 2.11% of total cells in control tissue and 1.65% in unruptured bAVMs, while Mϕ comprised 31.1% and 28.3%, respectively (Figure 2G and Supplemental Table 2), indicating no major shift in overall myeloid abundance. We next assessed cell type–specific expression of canonical myeloid activation markers. Within MG, *AIF1* expression was markedly increased in unruptured bAVMs compared with controls (median log-normalized expression 1.61 vs. 0.00, respectively; Figure 2H and Supplemental Table 3). A similar pattern was observed for *CD68*, with increased microglial expression in unruptured bAVMs relative to control tissue, despite overall low expression levels consistent with sparse transcription of lysosomal markers in resting MG (Figure 2I and Supplemental Table 4). In contrast, Mϕ exhibited more modest and heterogeneous expression changes. *AIF1* expression showed only a small difference between unruptured AVM and controls (median 1.39 vs. 1.10), while *CD68* expression remained low and did not demonstrate a consistent group-level increase (Supplemental Tables 5 and 6). The reanalyzed bulk RNA-seq and scRNA-seq allowed for histological comparison of MG/Mϕ in tissue specimens from STAs and unruptured bAVMs. Remarkably, we detected numerous Iba1^+^ and amoeboid shapes of CCR2^+^ cells in the unruptured bAVM tissue compared with the STA (Figure 2, B and C). Only a few MG/Mϕ were present during analysis of STA tissues, used as a control (Figure 2B). These findings support the hypothesis that MG/Mϕ seen in the bAVM territory are not merely presented as a result of vascular rupture.

### KRAS-ECs dynamically modulate MG/Mϕ in the bAVM territory.

**Figure 3:**
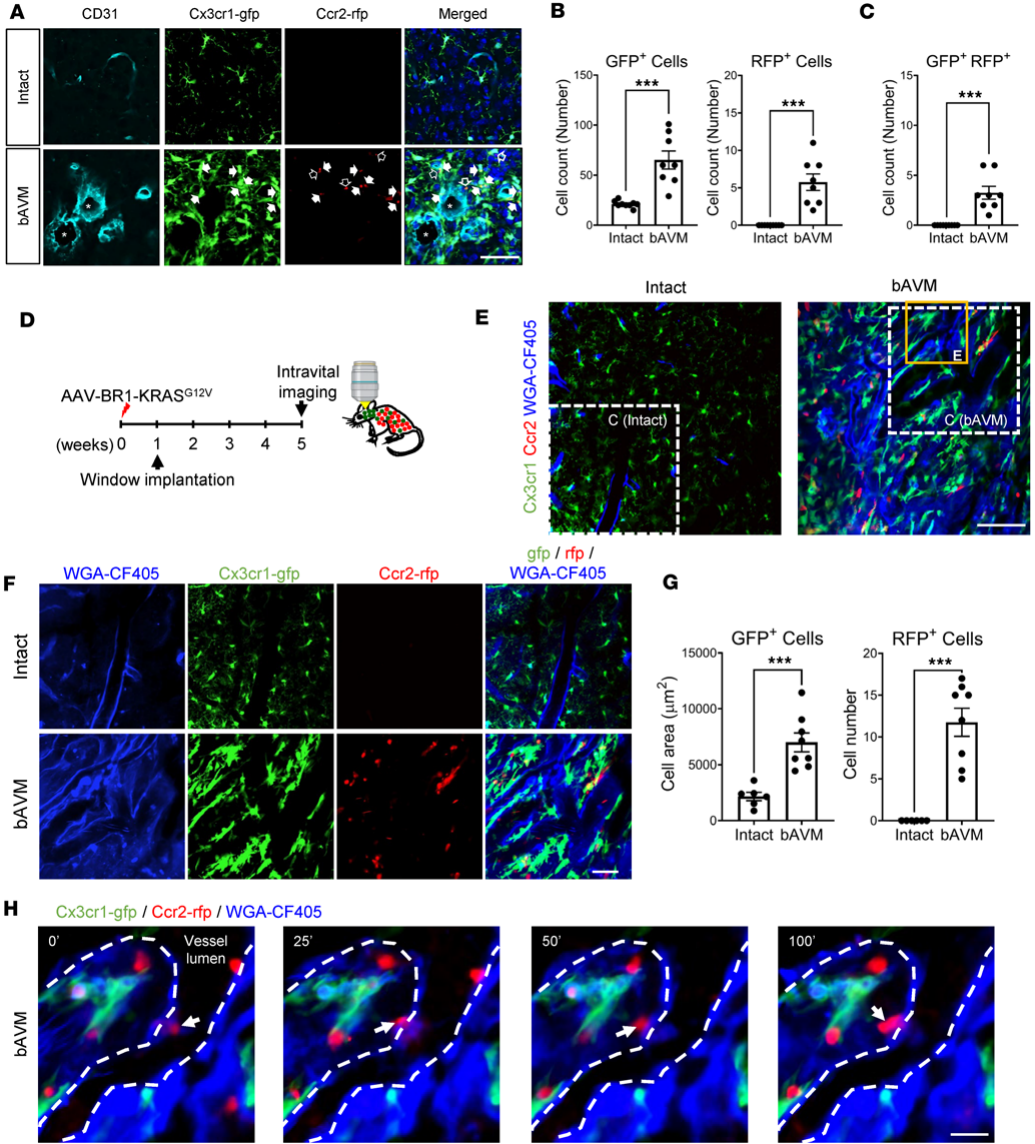
Intravital tracing of MG/Mϕ in bAVM territories in KRAS^G12V/bEC^ mice. (**A**) Representative immunofluorescence images showing the dense accumulation of both MG and Mϕ around bAVM vasculature. The dual-reporter *Cx3cr1-gfp;Ccr2-rfp* mice at 6 weeks after AAV-BR1-KRAS^G12V^ injection were used to visualize the spatial relationship between MG (CX3cr1-GFP, green), Mϕ (Ccr2-RFP, red), and vasculature (CD31; cyan) in bAVM territories. Note the enlarged lumens (asterisk) of vessels in the bAVM. Cx3cr1-GFP^+^ (green) microglia and Ccr2-RFP^+^ (red, arrow) Mϕ were more clustered around the bAVM territory, as compared with intact vessels. The arrows indicate colocalized activated Cx3cr1-GFP^+^ MG and infiltrated Ccr2-RFP^+^ blood-derived Mϕ at the enlarged bAVM vessels. Open arrow indicates Ccr2-RFP^+^ alone. Scale bar: 50 μm. (**B** and **C**) Bar graphs quantifying numbers of Cx3cr1-GFP^+^ MG or Ccr2-RFP^+^ Mϕ (**B**) and the coexpressed Cx3cr1-GFP^+^/Ccr2-RFP^+^ cells (**C**) around intact vessels versus bAVM-containing territories. Unpaired, 2-tailed *t* test. ***P < 0.001. Each dot indicates a randomly selected ROI (*n* = 8–9) obtained from mice (*n* = 3 per group). (**D**) A glass cranial window was implanted in the skull 1 week after AAV-BR1-KRAS^G12V^ injection in *Cx3cr1-gfp;Ccr2-rfp* mice. Four weeks later, intravital imaging was performed. (**E**) Representative immunofluorescence images indicating Cx3cr1-GFP^+^ MG and CCR2-RFP^+^ Mϕ around WGA-CF405^+^ dysplastic/malformed vessels in *Cx3cr1-gfp;Ccr2-rfp* mice. Scale bar: 100 μm. (**F**) Representative magnified images of white insets in **E** show the dense clustering of Cx3cr1-GFP^+^ MG and CCR2-RFP^+^ Mϕ in malformed vessels compared with intact. Scale bar: 50 μm. (**G**) Bar graphs quantifying numbers of Cx3cr1-GFP^+^ MG (GFP fluorescence pixel area) and Ccr2-RFP^+^ Mϕ (number of RFP^+^ cells) around dysplastic bAVM vessels compared to intact. All images were acquired by maximal projection. *Z*-stacks = 33 μm. Unpaired, 2-tailed *t* test. ***P < 0.001. Each dot indicates a randomly selected ROI (*n* = 6–8) obtained from mice (*n* = 3 per group). (**H**) Intravital time-lapse imaging captured from the inset (yellow box) in **E** shows the dynamic infiltration of Ccr2-RFP^+^ Mϕ from the dysplastic vessel lumen into the parenchyma. White arrows indicate the dynamic position of Ccr2-RFP^+^ cells (red). Dotted lines indicate the boundary between blood vessels and parenchyma. Scale bar: 20 μm.

To better understand bAVM-MG/Mϕ interactions in KRAS^G12C/bEC^ mice, we generated *Cx3cr1-gfp;Ccr2-rfp* dual-reporter bAVM mice, which allow MG (GFP, green) to be distinguished from blood-derived Mϕ (RFP, red) under fluorescence microscopy (31–33). Specifically, we injected AAV-BR1-KRAS^G12V^ into 6-week-old *Cx3cr1-gfp;Ccr2-rfp* mice. Six weeks later, we analyzed the distribution of MG/Mϕ around CD31^+^ malformed versus intact vessels. MG around bAVMs showed much thicker branches (Figure 3A), morphologically similar to those in Iba1 immunostaining (Figure 1B). Among numerous Cx3cr1-GFP^+^ MG, infiltrated Ccr2-RFP^+^ Mϕ were also detected in bAVM-affected brain parenchyma (Figure 3A). The number of Cx3cr1-GFP^+^ MG and Ccr2-RFP^+^ Mϕ was substantially higher in bAVM territory containing KRAS^G12V^-positive dysplastic vessels than in the intact region (non-bAVM region) (Figure 3B and Supplemental Figure 1, C–F). A particularly intriguing finding is the observation that coexpression of GFP^+^ MG and RFP^+^ Mϕ was observed around Ter-119^+^ ruptured bAVM territories in KRAS^G12V/bEC^ mice (Figure 3C and Supplemental Figure 2A). To further determine whether MG activation/Mϕ infiltration depends on the number of RBCs in malformed vessels, we compared the distribution and morphology of Cx3cr1-GFP^+^ MG and Ccr2-RFP^+^ Mϕ in the presence of high or low numbers of Ter-119^+^ RBCs. The numbers or morphology of Cx3cr1-GFP^+^ MG or Ccr2-RFP^+^ Mϕ were not dependent on RBC amount (Supplemental Figure 2, B and C). The results suggest that the activated MG and recruited Mϕ are not merely due to RBC extravasation in malformed vessels. Our data demonstrate that in KRAS^G12V/bEC^ mice, bAVMs induce activation of MG and recruitment of Mϕ, exacerbating inflammation in the bAVM territory.

Although there are studies that show monocyte infiltration into bAVMs (18, 19), the live-cell dynamics of MG activation/Mϕ recruitment into malformed vessels has not been studied. Using intravital analysis with *Cx3cr1-gfp;Ccr2-rfp* mice, we established myeloid cell tracing in KRAS^G12V^ mice at 5 weeks after bAVM induction. To visualize the dysplastic bAVM vessels, we injected wheat germ agglutinin (WGA)-CF405 (blue) (Figure 3D). Using this approach, the MG/Mϕ were readily observed in bAVM territories (Figure 3, E–G). MG (Cx3cr1-GFP^+^ cells) showed activated morphology, identified by their thick branches and enlarged soma. We also detected the presence of more round-looking Mϕ (Ccr2-RFP^+^ cells) that were located in parenchyma adjacent to bAVM vessels (Figure 3, F and H). Remarkably, this time-lapse approach was able to capture Mϕ extravasation at the site of the dysplastic vessels in bAVMs (Figure 3H and Supplemental Video 3). These data indicate that KRAS mutation–induced bAVM triggers the active recruitment of Mϕ from the circulation into bAVM territories. Notably, the intact WGA-CF405–labeled vessels exhibited a linear and continuous shape compared with the fragmented and tangled morphology of dysplastic bAVM vessels (34, 35) (Supplemental Figure 3), suggesting that the Cx3cr1-GFP^+^ MG and Ccr2-RFP^+^ Mϕ around the fragmented WGA-CF405–labeled vessels may contribute to BBB leakage.

Together, the results reported here indicate that the MG activation/Mϕ recruitment process may occur before hemorrhagic conversion, suggesting that MG/Mϕ-mediated inflammatory responses within bAVMs may precede bleeding.

### Inflammation associated with MG activation and Mϕ infiltration of bAVMs is a potential contributor to ICH in KRAS^G12V/bEC^ mice.

**Figure 4:**
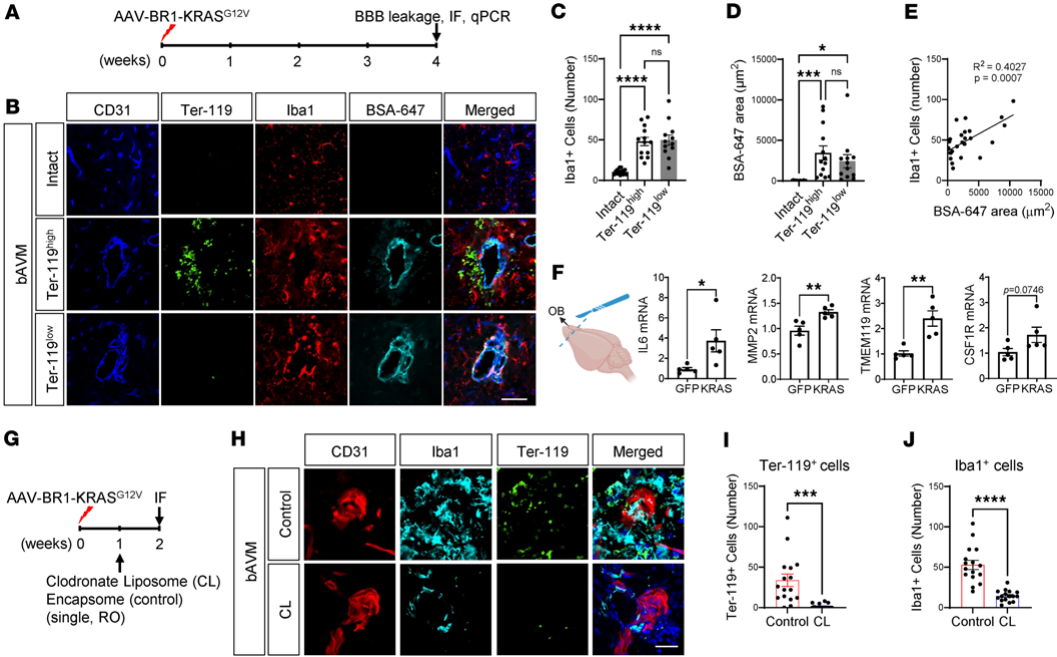
Early-stage–activated MG are responsible for the ICH occurrence in KRAS^G12V/bEC^ mice. (**A**) Brains were harvested 4 weeks after AAV-BR1-KRAS^G12V^ or AAV-BR1-eGFP (control) injection. (**B**) Representative immunofluorescence images showing robust Iba1^+^ MG and BSA-647 leakage in both ruptured (Ter-119^hi^) and unruptured (Ter-119^lo^) bAVM territory. Scale bar: 50 μm. (**C**–**E**) Bar graphs quantifying Iba1^+^ cell numbers (**C**) and area of BSA-647 leakage (**D**) in ruptured (Ter-119^hi^) and unruptured (Ter-119^lo^) bAVM territory compared with intact. Note that the BSA-647 leakage is correlated to the presence of Iba1^+^ cells (**E**). One-way ANOVA and Tukey’s multiple comparisons test and unpaired, 2-tailed *t* test. *P < 0.05; ***P < 0.001; ****P < 0.0001. NS, not significant. Each dot indicates a randomly selected ROI (*n* = 12–25) obtained from mice (*n* = 6 per group). (**F**) Bar graphs quantifying the increased mRNA levels of *IL6*, *MMP2*, *TMEM119*, or *CSF1R* in the dissected olfactory bulb (OB). Unpaired *t* test. *P < 0.05; **P < 0.01. Each dot indicates an individual mouse (*n* = 5 per group). The scheme was created with BioRender.com. (**G**) Clodronate liposomes (CLs) or control liposomes (Encapsome) were administered 1 week after AAV-BR1-KRAS^G12V^ injection. Brains were harvested 2 weeks after AAV-BR1-KRAS^G12V^ injection. (**H**) Representative immunofluorescence images showing reduced Iba1^+^ cells and Ter-119^+^ RBCs in CL-treated KRAS^G12V/bEC^ mice compared with control liposome–treated (Encapsome-treated) mice. Scale bar: 25 μm. (**I** and **J**) Bar graphs quantifying Ter-119^+^ RBCs in the parenchyma (**I**) and Iba1^+^ cells (**J**) in bAVM territories. Unpaired, 2-tailed *t* tests. ***P < 0.001, ****P < 0.0001. Each dot indicates a randomly selected ROI (*n* = 16) obtained from mice (*n* = 6 and 5 per group).

The results of intravital imaging, suggesting that MG activation and Mϕ infiltration of bAVMs precedes hemorrhagic conversion, are consistent with our previous observations demonstrating the presence of activated MG at some of the matured bAVMs, without signs of hemorrhagic conversion, at 9 weeks after AAV-BR1-KRAS^G12V^ injection (15). Here, at an even earlier 4-week time point after AAV-BR1-KRAS^G12V^, we found evidence of Iba1^+^ activated MG around unruptured dysplastic vessels, showing fewer parenchymal RBCs (Ter-119^lo^) as well as a markedly ruptured bAVM area (Ter-119^hi^) in the olfactory bulb (OB) (Figure 4, B and C). Interestingly, leakage of BSA-647, an indicator of BBB disruption, was still detected even in the unruptured bAVM area (Figure 4, B and D). Remarkably, the presence of Iba1^+^ cells correlated with the leakage of BSA-647 (Figure 4E).

To substantiate the claim that these inflammatory MG at the early stage of bAVM genesis could enhance inflammation, we assessed the expression of inflammatory mediators. Because most bAVMs are detected in the frontal lobe, including the OB (15), we selected the OB for inflammatory marker analysis. The analysis revealed that bAVM-containing OB tissue had increased expression of mRNAs encoding IL-6 (*IL6*), matrix metalloproteinase-2 (*MMP2*), TMEM119, and colony-stimulating factor 1 receptor (*CSF1R*) (MG/Mϕ activation/proliferation markers), as compared with the OB from the AAV-BR1-eGFP–injected (control) mice (Figure 4F). These data suggest that local inflammation at the site of bAVMs has the propensity to affect microvascular stability and lead to hemorrhagic rupture (36).

Therefore, to test whether the MG/Mϕ are indeed causally associated with hemorrhagic conversion of bAVMs causing ICH, we treated mice with CLs or control liposomes, starting 1 week after AAV-BR1-KRAS^G12V^ injection (Figure 4G), to deplete MG/Mϕ (37). One week later, reduced bleeding with CLs was achieved by direct quantification of RBCs in the parenchyma, where they surround the AVM vasculature, as demonstrated by immunofluorescent staining for the RBC marker Ter-119 (Figure 4, H and I). This improved vascular stability with CLs was accompanied by a reduced presence of Iba1^+^ cells around the malformed vessels, compared with control liposome–treated mice, which robustly showed Iba1^+^ cells with thick branches and enlarged soma (Figure 4, H and J). Furthermore, we observed a substantial reduction in T_2_*-MRI–detected hemorrhages using volumetric analysis with ITK-SNAP software (see Methods) and reduced Ter-119^+^ RBCs, accompanied by reduced Iba1^+^ cells by CL treatment (Supplemental Figure 4). These findings with CL treatment provide additional evidence linking MG/Mϕ to AVM instability/ICH.

### Depletion of MG/Mϕ attenuates hemorrhagic conversion in mice.

**Figure 5:**
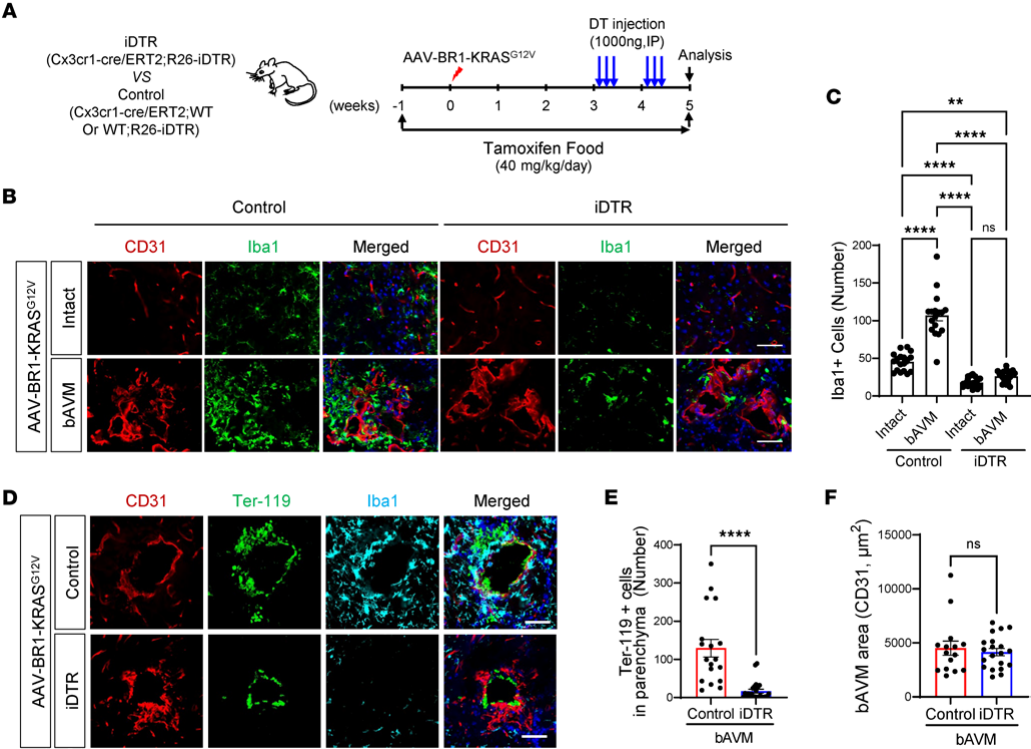
Genetic depletion of MG/Mϕ attenuates ICH in KRAS^G12V/bEC^ mice. (**A**) iDTR mice (*Cxcr1-cre/ERT2;Rosa26-iDTR*) and control mice (*Cxcr1-cre/ERT2*;WT or WT;*Rosa26-iDTR*) mice were fed with tamoxifen containing food starting 1 week before injection of AAV-BR1-KRAS^G12V^. To deplete the Cx3cr1^+^ MG/Mϕ, diphtheria toxin (DT) was administered 3 weeks and 4 weeks after AAV-BR1-KRAS^G12V^ injection. (**B**) Representative immunofluorescence images showing reduced Iba1^+^ MG and Mϕ in intact and bAVM territory in iDTR mice compared with control. Scale bar: 50 μm. (**C**) Bar graph quantifying number of Iba1^+^ cells in control and iDTR mice. One-way ANOVA with Tukey’s multiple comparisons test. **P < 0.01; ****P < 0.0001. NS, not significant. Each dot indicates a randomly selected ROI (*n* = 17–21) obtained from mice (*n* = 6–8 per group). (**D**) Representative immunofluorescence images showing reduced Ter-119^+^ RBCs and Iba1^+^ cells in bAVM territory from iDTR mice compared with control. Scale bars: 50 μm. (**E** and **F**) Bar graphs quantifying infiltrated Ter-119^+^ RBCs (**E**) and CD31^+^ bAVM area (**F**) in control and iDTR mice. Unpaired, 2-tailed *t* test. **P < 0.01, ****P < 0.0001. Each dot indicates a randomly selected ROI (*n* = 15–24) obtained from mice (*n* = 6–8 per group).

To further test whether MG/Mϕ link the bAVM instability and ICH occurrence, we utilized tamoxifen- and diphtheria toxin–inducible (DT-inducible) iDTR mice, which specifically deplete MG/Mϕ (38). To minimize the MG/Mϕ repopulation, DT was repeatedly administered on weeks 3 and 4 and mice euthanized within 4 days of the last injection (Figure 5A). Five weeks after AAV-BR1-KRAS^G12V^ injection, the iDTR mice treated with tamoxifen/DT showed a marked reduction in Iba1^+^ cells in both intact and bAVM areas compared with control mice (Figure 5, B and C). Remarkably, the number of Iba1^+^ cells was not substantially different between intact and bAVM areas in iDTR mice (Figure 5C). Notably, the infiltration of Ter-119^+^ RBCs into the parenchyma was substantially attenuated, while the CD31^+^ bAVM areas showed no difference in the iDTR mice compared with control (Figure 5, D–F). Our data show that MG/Mϕ specifically contribute to hemorrhagic conversion in the bAVM area.

### MG/Mϕ dynamically respond to antiinflammatory strategies in KRAS^G12V/bEC^ mice.

**Figure 6:**
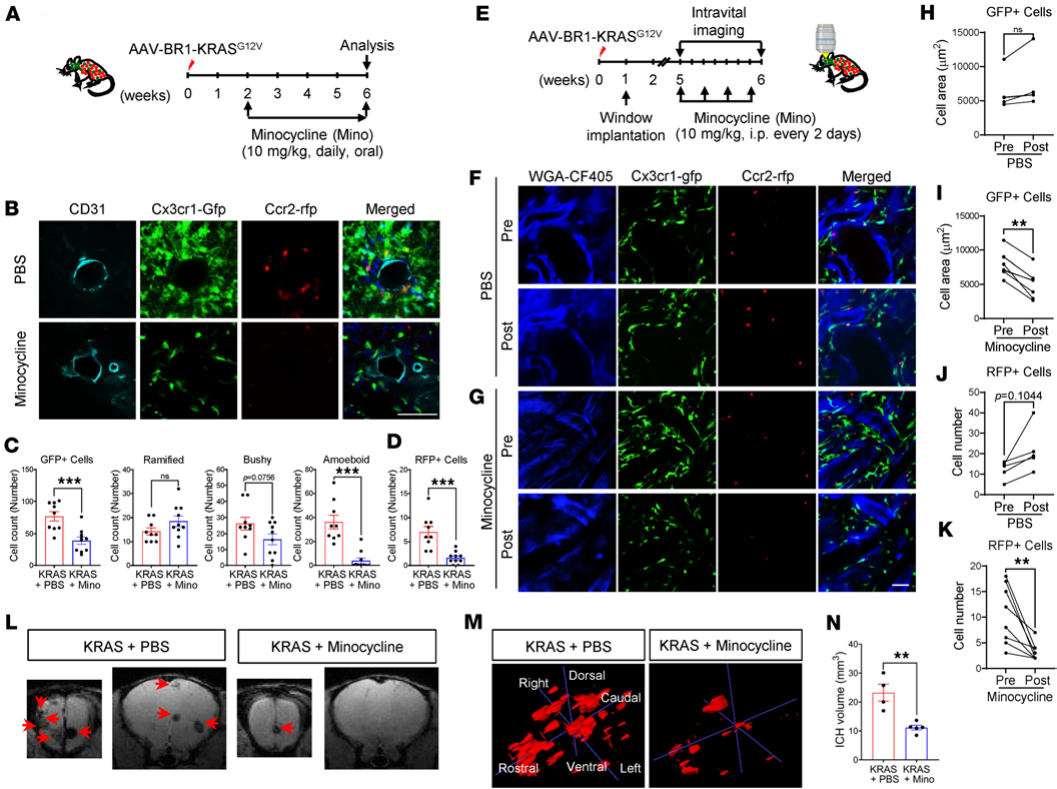
Minocycline reduces activation of MG and recruitment of Mϕ toward bAVM territories in KRAS^G12V/bEC^ mice. (**A**) KRAS^G12V/bEC^ mice received daily minocycline for 4 weeks starting 2 weeks after AAV-BR1-KRAS^G12V^ injection, and then mice were imaged with T_2_*-weighted MRI and brains were analyzed at 6 weeks. (**B**) Representative immunofluorescence images showing attenuated clustering of Cx3cr1-GFP^+^ MG and Ccr2-RFP^+^ Mϕ around CD31^+^ (vessel, cyan) bAVM territory in KRAS^G12V/bEC^ mice treated with minocycline compared with PBS. Scale bar: 50 μm. (**C** and **D**) Bar graphs quantifying numbers of Cx3cr1-GFP^+^ MG for total, ramified, bushy, and amoeboid based on morphology assessment (**C**) or total numbers of Ccr2-RFP^+^ Mϕ (**D**) in the bAVM territory between KRAS^G12V/bEC^ mice treated with minocycline or PBS. Unpaired, 2-tailed *t* test. ***P < 0.001. Each dot indicates a randomly selected ROI (*n* = 9–10) obtained from mice (*n* = 5 per group). (**E**) KRAS^G12V/bEC^ mice received minocycline once every 2 days, 4 times in total, starting 5 weeks after AAV-BR1-KRAS^G12V^ injection. The mice were observed with intravital imaging before and after the treatment. (**F** and **G**) Representative immunofluorescence images showing reduced clustering of Cx3cr1-GFP^+^ MG and Ccr2-RFP^+^ Mϕ in WGA-CF405–labeled dysplastic bAVM vessels in KRAS^G12V/bEC^ mice treated with minocycline (**G**) compared with PBS (**F**). Scale bar: 50 μm. (**H**–**K**) Bar graphs quantifying Cx3cr1-GFP^+^ MG (**H** and **I**, fluorescent pixel area) and Ccr2-RFP^+^ Mϕ (**J** and **K**, count of RFP^+^ cells) around dysplastic bAVM vessels. Individual ROIs were compared between pre- and posttreatment results for PBS- and minocycline-treated groups, respectively. A traced ROI compared between time points highlights the rapid reduction of GFP- and RFP-expressing cells following treatment with minocycline. Unpaired, 2-tailed *t* test. **P < 0.01. Each dot indicates a randomly selected ROI (*n* = 5–8) obtained from mice (*n* = 3 per group). (**L** and **M**) Representative T_2_*-weighted MRI (**L**) and ITK-SNAP volumetric images (**M**) show that minocycline reduced the ICH lesions in KRAS^G12V/bEC^ mice. (**N**) Bar graphs quantifying ICH volume. Unpaired, 2-tailed *t* test. **P < 0.01. Each dot indicates the hemorrhagic volume of an individual mouse (*n* = 4–5 per group). NS, not significant.

An abundance of MG (Cx3cr1-GFP^+^ cells) was present at bAVM locations (Figure 3A). They displayed heterogeneous morphologies, including ramified (quiescent), bushy (intermediate activation), and amoeboid (activated and phagocytic) shapes (15, 39). This diversity in MG morphology is a key indicator of their activation stage. Notably, we found a high number of MG showing activated morphology at the bAVM location (Figure 6, B and C). Using this approach of MG activity assessment, we tested whether the destabilization of malformed vessels from early to mature stages of bAVM growth, which could lead to ICH, involved changes in MG activation and Mϕ infiltration. We induced bAVM in *Cx3cr1-gfp;Ccr2-rfp* mice and then 2 weeks later (to allow time for minocycline to interfere with bAVM formation) treated them with antiinflammatory minocycline (40) (Figure 6A). After 4 weeks of treatment, we found that minocycline substantially attenuated the activation of MG (Cx3cr1^+^ cells) and reduced infiltration of Mϕ (Ccr2^+^ cells) (Figure 6B). The total number of MG/Mϕ was lower at the bAVMs of minocycline-treated groups (Figure 6, C and D). The number of amoeboid-shaped MG, indication their phagocytic activation, was drastically reduced by minocycline, while the number of ramified nonactivated MG was unchanged (Figure 6C). Our data provide evidence that minocycline reduces inflammation in bAVMs through inhibiting MG activation and reducing Mϕ recruitment to the bAVM area. Moreover, using direct histology, we quantified MG/Mϕ using an intravital imaging approach with a glass window implanted in skulls of the *Cx3cr1-gfp;Ccr2-rfp*/AAV-BR1-KRAS^G12V^ mice. To study the effect of minocycline on the fate of mature bAVMs in this experiment, we initiated minocycline treatment 5 weeks after induction of bAVMs with AAV-BR1-KRAS^G12V^ (Figure 6E). Examination of these mice revealed that activated MG (GFP^+^/green cells having thick branches with enlarged soma) and infiltrated Mϕ (RFP^+^/red, round-shaped cells) were abundant in bAVM territories 5 weeks after bAVM induction (Figure 6, F and G). Remarkably, only 1 week of treatment with minocycline was sufficient to reduce the abundance of bAVM-associated MG/Mϕ in these mice (Figure 6, G–I). It is important to note that animals treated with PBS, instead of minocycline, showed increased MG numbers and Mϕ infiltration, suggesting an ongoing progression of inflammation in the bAVM territory between weeks 5 and 6 (Figure 6, F, H, and I).

### Inactivating MG/Mϕ attenuates hemorrhagic conversion in the destabilized, malformed vessels of KRAS^G12V/bEC^ mice.

Next, we probed whether inhibition of MG activation and Mϕ infiltration with minocycline reduces hemorrhagic conversion of bAVMs. We treated mice with minocycline from 2 to 6 weeks after AAV-BR1-KRAS^G12V^ injection and quantified the volume of ICHs at the end of minocycline treatment using T_2_*-weighted MRI imaging and ITK-SNAP software (Figure 6L). Strikingly, minocycline robustly reduced bAVM-associated hemorrhages and ICH volume compared with PBS-treated mice (Figure 6, L–N, and Supplemental Figure 5), indicating that treatment with minocycline during bAVM formation potently attenuated hemorrhagic conversion.

**Figure 7:**
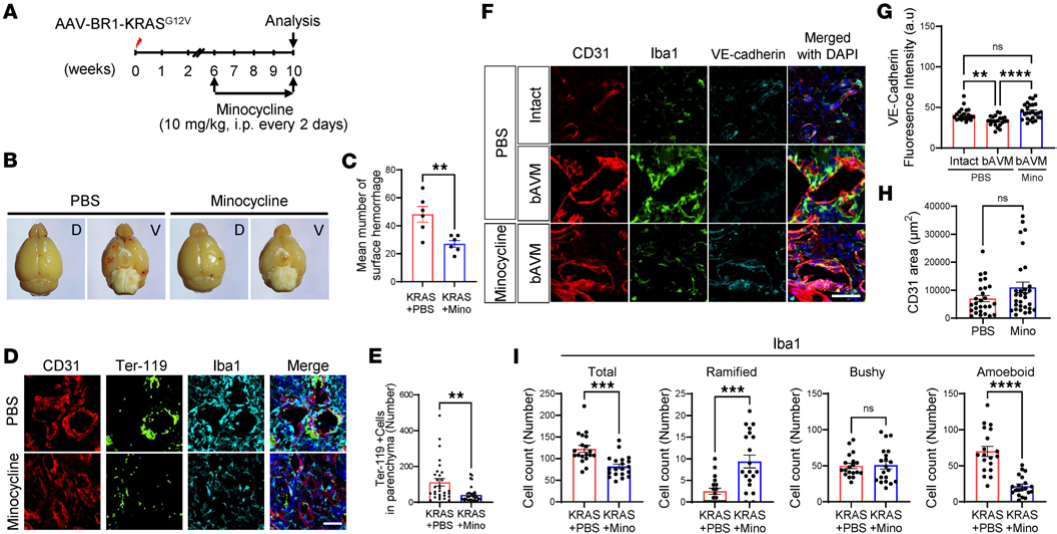
Posttreatment with minocycline attenuates ICH in KRAS^G12V/bEC^ mice. (**A**) KRAS^G12V/bEC^ mice received minocycline for 4 weeks, every 2 days, starting from 6 weeks after AAV-BR1-KRAS^G12V^ injection, and the brains were analyzed at 10 weeks. (**B** and **C**) Representative images showing surface hemorrhages in KRAS^G12V/bEC^ mice treated with PBS or minocycline (**B**). D indicates dorsal view. V indicates ventral view. (**C**) Bar graphs quantifying the number of surface hemorrhages. Unpaired, 2-tailed *t* test. *P < 0.05. Each dot indicates the mean number of surface hemorrhages from an individual mouse (*n* = 6 per group). (**D** and **E**) Representative immunofluorescence images showing reduced Ter-119^+^ RBCs and Iba1^+^ MG/Mϕ in KRAS^G12V/bEC^ mice receiving posttreatment minocycline compared with PBS (**D**). Scale bar: 50 μm. (**E**) Bar graphs quantifying the number of Ter-119^+^ RBCs in the parenchyma. Unpaired, 2-tailed *t* test. **P < 0.01. Each dot indicates a randomly selected ROI (*n* = 30–34) from mice (*n* = 6–7 per group). (**F**) Representative immunofluorescence images showing reduced Iba1^+^ MG/Mϕ and restored VE-cadherin. Scale bar: 50 μm. (**G** and **H**) Bar graphs quantifying VE-cadherin^+^ fluorescence intensity (**G**) and CD31^+^ vessel area (**H**). **P < 0.01; ****P < 0.0001 by 1-way ANOVA with Tukey’s multiple comparisons test (**G**) or unpaired, 2-tailed *t* test (**H**). Each dot indicates a randomly selected ROI (**G**, *n* = 21–26; **H**, *n* = 27–30) obtained from mice (*n* = 6–7 per group). (**I**) Bar graphs quantifying numbers of Iba1^+^ MG/Mϕ for total, ramified, bushy, and amoeboid based on morphology assessment in the bAVM territory between KRAS^G12V/bEC^ mice treated with minocycline or PBS. Unpaired, 2-tailed *t* test. *P < 0.05, ***P < 0.001. Each dot indicates a randomly selected ROI (*n* = 19–20) obtained from mice (*n* = 6–7 per group). NS, not significant.

Although the data with minocycline during bAVM development provide conceptual evidence for a pivotal role for MG/Mϕ in the destabilization of growing bAVMs, the more therapeutically relevant approach is to establish whether minocycline could stabilize and prevent hemorrhagic conversion of mature bAVMs, similar to what we tested with intravital analysis (Figure 6, E–K). Thus, 6 weeks after AAV-BR1-KRAS^G12V^ injection, KRAS^G12V/bEC^ mice were treated with minocycline, a time point when bAVMs are typically matured and exhibit hemorrhages (Figure 7A). After 4 weeks of treatment with minocycline or PBS every other day, this delayed treatment with minocycline substantially attenuated vascular disruption, as demonstrated by reduced visual morphological evidence of brain surface hemorrhages (Figure 7, B and C) and histological evidence of bleeding (Ter-119^+^ RBCs), compared with mice receiving PBS (Figure 7, D and E). The vascular disruption, indicated by the loss of BBB genes, was tested by identifying the decreased gene levels of BBB junction markers, including *CDH5*, *TJP1*, *OCLN*, and *CLDN5* in unruptured human bAVM tissues using a published scRNA-seq dataset (30) (Supplemental Figure 6). To further validate the BBB loss in KRAS^G12V/bEC^ mice, we established loss of vascular integrity by showing the loss of VE-cadherin (CDH5) on the CD31^+^ malformed vessels and demonstrated that treatment with minocycline attenuated this VE-cadherin loss (Figure 7, F and G). Importantly, KRAS^G12V/bEC^ mice receiving minocycline or PBS showed a similar abundance of CD31, suggesting that minocycline treatment at later stages has no effect on further bAVM growth (Figure 7, F and H). Notably, the attenuation of hemorrhagic events and the preservation of endothelial VE-cadherin were associated with the attenuation of MG activation and Mϕ infiltration within bAVM territories (Figure 7I).

**Figure 8:**
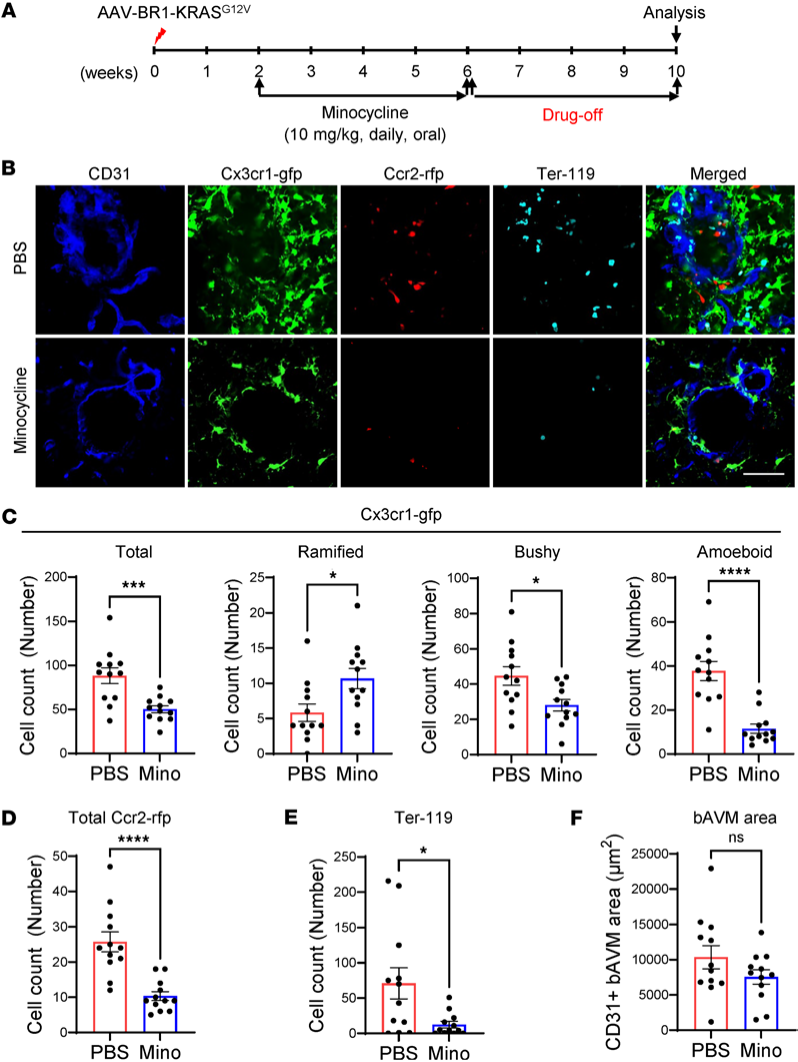
Minocycline exerts a sustained inhibitory effect on MG/Mϕ activation/infiltration in bAVMs following treatment cessation. (**A**) Male *Cx3cr1-gfp/Ccr2-rfp* mice daily received oral administration of minocycline from 2 to 6 weeks after AAV-BR1-KRAS^G12V^ injection, followed by drug withdrawal (drug-off) for 4 weeks. (**B**) Representative immunofluorescence images showing attenuated clustering of Cx3cr1-GFP^+^ MG and Ccr2-RFP^+^ Mϕ, and Ter-119^+^ RBCs around CD31^+^ (vessel, cyan) bAVM territory in KRAS^G12V/bEC^ mice treated with minocycline compared with PBS. Scale bar: 50 μm. (**C**–**F**) Bar graphs quantifying numbers of Cx3cr1-GFP^+^ MG for total, ramified, bushy, and amoeboid based on morphology assessment (**C**), total numbers of Ccr2-RFP^+^ Mϕ (**D**), numbers of infiltrated Ter-119^+^ RBCs in parenchyma (**E**), or area of CD31^+^ bAVMs (**F**) in the bAVM territory between KRAS^G12V/bEC^ mice treated with minocycline or PBS. Unpaired, 2-tailed *t* test. *P < 0.05, ***P < 0.001, ****P < 0.0001. Each dot indicates a randomly selected ROI (*n* = 12) from mice (*n* = 6 per group). NS, not significant.

Finally, we tested whether minocycline treatment maintains a long-lasting effect on the attenuated hemorrhagic conversion in bAVM mice. *Cx3cr1-gfp;Ccr2-rfp* mice injected with AAV-BR1-KRAS^G12V^ were treated with minocycline from 2 to 6 weeks, followed by a 4-week drug-off period (Figure 8A). Ten weeks after AAV-BR1-KRAS^G12V^ injection, the bAVM area displayed that MG are less activated by exhibiting the lower bushy/activated Cx3cr1-GFP^+^ MG and a reduced number of infiltrated Ccr2-RFP^+^ Mϕ (Figure 8, B–D), accompanied by fewer Ter-119^+^ RBCs than the PBS-treated group (Figure 8E). The CD31^+^ bAVM area did not change after the drug-off period (Figure 8F). Additionally, there were no sex differences in the numbers of Cx3cr1-GFP^+^ MG and Ccr2-RFP^+^ Mϕ, RBCs, and CD31^+^ bAVM areas in male and female bAVM mice that received either PBS or minocycline (Supplemental Figure 7). Furthermore, the drug-off period between 10 and 14 weeks after minocycline treatment from 6 to 10 weeks showed a substantial attenuation of RBC infiltration and Iba1^+^ cell activation/infiltration (Supplemental Figure 8). The data suggest that the drug-off period does not affect the return of MG activation/Mϕ infiltration and maintains a long-lasting effect in attenuating hemorrhagic conversion. Regarding whether minocycline could reduce systolic blood pressure (41), the measurement of chronic blood pressure of KRAS^G12V/bEC^ mice treated with minocycline did not show a substantial difference compared to the control (Supplemental Figure 9 and Supplemental Table 7). Taken together, these results suggest that treatment with minocycline, through inhibition of MG activation and Mϕ infiltration, may stabilize bAVMs and reduce further ICH risk.

## Discussion

Hemorrhagic conversion in malformed vessels causes neurological disability or death in patients with bAVM. The mechanism driving ICH in patients with bAVM has long been considered to be the result of high blood pressure and shearing injury against weakened vessels due to vascular shunting (42–44). However, the evidence confirming the mechanisms underlying AVM destabilization and leading to ICH is scarce. In this study, we examined a mechanism of bAVM destabilization and ICH by exploring the role of MG/Mϕ in the bAVM territory using a human-relevant sporadic bAVM mouse model. We observed that selective overexpression of mutant KRAS^G12V^ in bECs (to model human bAVM pathology) caused dysplastic changes similar to human bAVM and it results, similar to what we have observed for human bAVM tissue, in activation/recruitment of MG/Mϕ to the malformed vessels. This recruitment of immune cells to bAVM sites preceded hemorrhagic conversion and their inhibition attenuated this hemorrhagic conversion, suggesting a causal association between activated MG/infiltrated Mϕ and bAVM-associated ICH. Our results imply that exacerbated inflammation around bAVM vessels compromises vascular integrity and increases the risk for spontaneous ICH, suggesting that inhibition of this process could represent a therapeutic target for reducing ICH risk in patients with bAVM.

MG/Mϕ are major inflammatory cell populations that have been previously reported in human bAVM tissues and preclinical HHT mouse models (18, 19, 30, 45). Likewise, we previously reported observations that KRAS^G12V/bEC^ mice exhibit inflammatory features, including dynamically morphing myeloid cells (mainly activated MG and infiltrated Mϕ) in corresponding areas of robust bleeding (15). In bAVM territories (exhibiting dysplastic vessels with a nidus of AVM), MG/Mϕ displayed a higher prevalence of bushy or amoeboid morphologies, associated with activated MG and infiltrating Mϕ, which was markedly different from the intact areas. The increased presence of MG/Mϕ at ruptured mouse and human AVMs could drive normal repair mechanisms, as MG/Mϕ are typically recruited to repair ruptured cerebral vessels and clear hemorrhagic debris (46–49). However, our observation of an increased presence of MG/Mϕ at unruptured bAVM territories suggests that the activated MG/infiltrated Mϕ have a more complex role to play than to clear and repair the damage. Moreover, the prevalence of MG/Mϕ around unruptured hemosiderin-negative human bAVM tissues corroborated with MG/Mϕ presence in human bAVM tissue (50), suggesting that the activation of brain MG and recruitment of monocyte-derived Mϕ into bAVM vessels may indeed contribute to vascular disruption and extravasation of erythrocytes.

To better understand the inflammatory process in bAVMs, we used *Cx3cr1-gfp:Ccr2-rfp* dual-reporter mice that permitted us to distinguish between activated Cx3cr1-GFP^+^ MG and infiltrated Ccr2-RFP^+^ Mϕ within bAVM territories. The chemokine receptor Cx3cr1 is a mediator of chemotaxis for immune cells and is expressed on several cell types within the immune system, including Mϕ, MG, T cells, natural killer (NK) cells, and dendritic cells (51–53). However, Cx3cr1 expression on the different cell types is largely tissue dependent. In the CNS, preferential expression of Cx3cr1 has been observed on MG. The chemokine receptor Ccr2 is expressed on immune cells in the peripheral blood, including monocytes, immature dendritic cells, activated T cells, and B cells (54). Importantly, it has been shown that MG do not express Ccr2 from embryonic development to adulthood (55). By evaluating the relative expression of Cx3cr1 to Ccr2, a clear distinction can be made between infiltrating Mϕ and resident MG (33). Interestingly, Cx3cr1-GFP and Ccr2-RFP were coexpressed in bAVM territories in our bAVM mouse model (Figure 3, A and C). Although the expression levels of Cx3cr1 and Ccr2 in those cells need to be determined, the GFP^+^/RFP^+^ cells suggest the possibility of diverse phenotypes, including Cx3cr1^hi^Ccr2^lo^ cells that patrol noninflamed areas and Ccr2^hi^Cx3cr1^lo^ cells with more proinflammatory roles (56–58). Ccr2-RFP^+^ monocytes could transit to become resident MG after hypoxic injury, suggesting the GFP^+^/RFP^+^ cells show the monocyte-to-MG transition during hemorrhagic conversion (59). In addition, GFP^+^/RFP^+^ cells were often found where RBC infiltration was high (Supplemental Figure 2). This suggests that Cx3cr1^+^, Ccr2^+^, or GFP^+^/RFP^+^ cells, together, could exacerbate inflammation (60, 61), thereby eliciting ICH in the bAVM territory. The dynamic movement of Cx3cr1^+^ MG and Ccr2^+^ Mϕ was further investigated using an intravital imaging approach. We observed that Ccr2^+^ Mϕ enter malformed blood vessels from the systemic circulation. Furthermore, the Ccr2^+^ Mϕ migrated to areas where Cx3cr1^+^ MG exhibiting thick branches and soma preexisted around dysplastic vessels (Figure 3H). This suggests that the process driving Mϕ infiltration may be associated with interactions between the cells comprising dysplastic malformed vessels, including KRAS-ECs or activated MG (62). Further investigation is required to uncover the mechanisms by which mutant KRAS–ECs interact with activated MG and infiltrated Mϕ.

Meanwhile, existing studies suggest that activated MG/infiltrated Mϕ may incite local inflammation, which disrupts the BBB and weakens the vasculature (63, 64). In experimental stroke models, the loss of junction proteins, particularly VE-cadherin (CDH5), was linked to the presence of inflammatory mediators (65, 66). Our data demonstrating increased production of proinflammatory IL-1β and IL-6 in MG/Mϕ (Figure 1, G–J) and decreased VE-cadherin expression on the bAVM vessels surrounded with MG/Mϕ (Figure 7, F and G) in KRAS^G12V/bEC^ mice and increased inflammatory genes (*CD68* and *AIF1*) in MG (45, 67) and decreased junctional marker genes in the unruptured human bAVM tissues (30) (Figure 2 and Supplemental Figure 6) suggests simultaneous loss of BBB integrity due to MG/Mϕ-mediated inflammation in the bAVM territory. Our study has taken the initial steps toward understanding the role of MG/Mϕ in the ruptured and unruptured bAVM territories.

Our findings of activated MG/infiltrated Mϕ in unruptured bAVM regions in combination with upregulation of various inflammatory genes at an early time point (Figure 4) suggests that MG/Mϕ play a pivotal role in driving bAVM instability through local inflammatory processes. This claim is supported by our results showing that the early depletion of MG, using CLs, attenuates RBC extravasation and ICH. The data showing that round-shaped Iba1^+^ cells (suggestive of Mϕ) were not detected in bAVM territories 2 weeks after AAV-BR1-KRAS^G12V^ injection (Figure 4H) suggests that the early activation of MG may be an initiating factor in hemorrhagic conversion of these vessels. Likewise, a study with an earlier MG/Mϕ depletion using the CSF1R inhibitor PLX5622 also showed reduced dysplastic vessels, hemorrhages, and increased claudin 5 expression in focal Alk1-depleted bAVM mice, similar to our current study (19). Importantly, the controlled transgenic approach depleting MG/Mϕ using iDTR mice between 3 and 5 weeks remarkably attenuated hemorrhagic conversion. The time window between the last DT injection and brain harvesting was within 4 days; thus, this approach excludes the possibility of repopulated MG (38). Moreover, regarding Mϕ, which usually lose the DTR by 3 weeks after the last tamoxifen injection (68), our experiments included Mϕ depletion, as tamoxifen-induced DTR labeling on Mϕ persists until the mice are euthanized after receiving the repeated DT injections. Thus, the approach allows for the depletion of both MG and Mϕ, and the data suggest a specific requirement for MG/Mϕ in hemorrhagic conversion. This overall suggests that MG activation/Mϕ infiltration in the bAVM territory creates a local inflammatory environment that disrupts vascular integrity.

Our further study using minocycline provides additional evidence to support the claim that targeting MG activation/Mϕ infiltration may be an effective strategy for attenuating hemorrhagic conversion of malformed vessels. Owing to minocycline’s antibiotic properties (69) along with its high CNS penetrance and reported antiinflammatory mechanisms, minocycline has gained recognition for its antineuroinflammatory properties that prove effective in a wide variety of neurological diseases (70, 71). Minocycline has also been reported to effectively reduce activation/recruitment of MG/Mϕ in a mouse model of subarachnoid hemorrhage (72), mitigate ICH in patients with severe cerebral amyloid angiopathy, and reduce BBB loss following hemorrhagic stroke (73, 74). Here, we capitalized on these antiinflammatory properties and demonstrated that minocycline reduced the severity and frequency of ICHs in KRAS^G12V/bEC^ mice. Importantly, we showed that late treatment with minocycline starting 6 weeks after AAV-BR1-KRAS^G12V^ injection, which corresponds to mature bAVM development, also resulted in inhibited MG activation/Mϕ infiltration, prevented loss of junction proteins, and attenuated hemorrhagic conversion. This suggests that minocycline as a broad antiinflammatory agent may hold benefits for patients with bAVM at high risk for hemorrhagic conversion. However, minocycline has side effects, including gut microbiome disruption and immunosuppressive activity (75, 76). However, the antiinflammatory activities of minocycline are reported beyond its antimicrobial effect in infectious diseases (77). Notably, the long-lasting effect of minocycline after treatment cessation attenuated hemorrhagic conversion, accompanied by reduced inflammatory MG/Mϕ. Given the potential side effects of long-term minocycline treatment, the results of this drug-off experiment suggest that modulating the treatment regimen could minimize these side effects, highlighting the benefits of reducing minocycline-induced inflammation. Thus, clinical trials are needed to further determine the dose efficacy and safety of long-term minocycline use in patients with bAVM.

Patients with bAVM most commonly experience hemorrhages as young adults between the ages of 20 and 30, which corresponds to 3–6 months of age in mice (78, 79). In our model, we observed ICH as early as 2 weeks and substantial ICH by 6 weeks after AVM induction, suggesting a more aggressive pathology than in the human bAVM pathology. This exacerbation likely resulted from the AAV-based overexpression system, which differed from the *ibEC-KRAS^G12D^* mouse model, in which tamoxifen was administered at P1, and hemorrhages were rare by 8 weeks of age (16). Consequently, the aggravated hemorrhagic conversion in our bAVM mouse model represents a limitation and should be weighed against the extent to which the bAVM model recapitulates the mechanisms of human bAVM pathology. On the other hand, the pronounced and early-onset pathology in our mouse model enables robust investigation of bAVM development and ICH mechanisms in young-adult mice. These characteristics make our bAVM mouse model highly valuable for evaluating drug efficacy in preventing ICH and for determining the translational potential of pharmacological interventions.

Taken in context, our study showed that somatic mutation of KRAS in ECs activates and recruits MG/Mϕ toward bAVM territories, resulting in bAVM destabilization and an increased risk for bAVM rupture. Our study provides valuable evidence for inflammation-mediated pathophysiology of bAVM rupture/ICH. The findings may aid in the search for noninvasive therapeutic approaches for patients with bAVM.

## Methods

### Sex as a biological variable.

This study examined both male and female mice. There is no substantial difference in AVM formation, ICH occurrence, and MG activation/Mϕ infiltration between males and females; thus, sex was not considered as a biological variable in this study.

### Animals.

Male and female C57BL/6J mice (strain 000664), *Cx3cr1-gfp;Ccr2-rfp* dual-reporter mice (strain 032127) (32, 33), *Cx3cr1-cre/ERT2* (strain 020940), and *Rosa26-iDTR* (strain 007900) (38, 68) were purchased from The Jackson Laboratory. The mice were bred at the Center for Laboratory Animal Medicine and Care (CLAMC) at the University of Texas Health Science Center at Houston (UTHealth Houston) and validated with genotyping protocols provided by The Jackson Laboratory.

For depleting MG**/**Mϕ, 5-week-old iDTR mice received tamoxifen-containing food (40 mg/kg/day per mouse; TD.130860, Inotiv), followed by injection with AAV-BR1-KRAS^G12V^. Three weeks after AAV-BR1-KRAS^G12V^ injection, the mice received DT (1,000 ng/mouse; D0564, Sigma-Aldrich) for 3 consecutive days on weeks 3 and 4. Analysis was performed 5 weeks after AAV injection (Figure 5A). Age-matched 5- to 6-week-old male and female mice weighing 20–25 g were used for studying the MG/Mϕ dynamics in bAVM territories. The experimental group was randomly selected, and the data were analyzed by blinded researchers. All experimental mice were included in the analysis.

### AAV production and injection.

As previously described (29), we obtained pXX2-187-NRGTEWD (a plasmid harboring the AAV type 2 rep/cap genes with bEC-targeting peptide insertion, called AAV-BR1 cap) from the University Medical Center Hamburg-Eppendorf. For mouse experiments, the AAV-BR1-CAG-human (h) KRAS^G12V^-WPRE and -eGFP-WPRE (as a control) were produced by a custom AAV production service (Vector Biolabs). Six-week-old male or female mice were administered 100 μL of PBS (SH30256.01, Cytiva, Hyclone Laboratories) containing 1.25 × 10^10^ (for mouse brain clearing/3D immunostaining) or 2.5 × 10^10^ genome copies (GC)/mouse of AAV-BR1-CAG-hKRAS^G12V^-WPRE or -eGFP-WPRE by retro-orbital (RO) injection of the venous sinus under anesthesia (15, 80).

### Human bAVM tissue collection.

Human brain AVM tissues and control STA were dissected from patients with bAVM. The neurosurgeon collected the bAVM samples from the operating room after resection. Adverse events were not expected from bAVM sample collection, since the samples were only obtained from tissues that were normally extracted as part of the bAVM resection procedure. A small segment of the STA was collected, which was used for control. The STA samples were obtained from already-cut vessels, which had been exposed as part of the scalp incision associated with the bAVM resection procedure. Removing small segments from these already-cut vessels does not lead to any appreciable change in blood supply or tissue integrity for healing. The samples were labeled and stored at –80°C at the UTHealth Houston McGovern Medical School.

### Analyzing bulk RNA-seq and scRNA-seq data.

Bulk RNA-seq and scRNA-seq data were obtained from publicly available datasets: dbGaP (accession: phs002624.v2.p1), which includes ruptured bAVM, unruptured bAVM, and control adult cortex vessels from epilepsy patients, originally published by Winkler et al. (30). For bulk RNA-seq analysis, expression levels of genes were compared between ruptured and unruptured bAVM tissues, with a focus on genes associated with MG/Mϕ. For scRNA-seq analysis, preprocessing, quality control, normalization, dimensionality reduction, clustering, and cell-type annotation were performed by strictly following the analytical pipelines and criteria described in Winkler et al. (30). Immune and vascular cells, including MG/Mϕ, were identified using the same marker-based definitions and annotation strategies reported in Winkler et al. (30). For immune cells, cell-type proportions were calculated at the sample level. Proportions of MG and Mϕ were compared between experimental groups using nonparametric statistical tests. Targeted gene expression analyses focused on *AIF1* and *CD68*. Expression levels were evaluated within MG and Mϕ separately and compared across unruptured AVM and control samples. For vascular cells, the expression levels of genes involved in EC junction integrity were examined. Specifically, the junction-associated genes examined were *CDH5*, *TJP1* (ZO-1), *OCLN*, and *CLDN5*. To assess cell proportions, binary classification (expression >0) was applied, and Fisher’s exact tests were used to determine statistical significance. All analyses were conducted in R version 4.3 (https://www.R-project.org). Expression levels of selected genes were extracted across annotated cell types, and Wilcoxon’s rank-sum tests were used to compare unruptured AVM versus control conditions.

### Treatment with CLs and minocycline.

To deplete Mϕ, C57BL6/J mice were treated with CLs (CLD-8909) or control liposomes (Encapsome, CLD-8910, Encapsula Nanoscience LLC; 1 mg/20 g mouse, single, RO) at 1 week after AAV-BR1-KRAS^G12V^ injection. One week after CL or control liposome treatment, the mice were scanned using T_2_/T_2_*-MRI, and ICH volumes were measured from MRI imaging using ITK-SNAP 4.0.2 (https://www.itksnap.org/pmwiki/pmwiki.php). Minocycline hydrochloride (M9511, MilliporeSigma) was prepared with PBS (0.05 mg/mL) and stored at –20°C. The *Cx3cr1-gfp;Ccr2-rfp* mice were treated with daily minocycline (10 mg/kg/day, oral treatment, daily) or saline (as a control) for 4 weeks, which started 2 weeks after AAV-BR1-KRAS^G12V^ injection (Figure 6A and Figure 8). The mice were scanned using T_2_/T_2_*-weighted MRI or magnetic resonance angiography (MRA) before (at 2 weeks) or after (at 6 weeks) minocycline treatment (Supplemental Figure 5). For intravital imaging testing of the changes in MG/Mϕ in the short-term study (Figure 6E), *Cx3cr1-gfp;Ccr2-rfp* mice received minocycline via intraperitoneal injection to increase its bioavailability versus other administration routes (81). The *Cx3cr1-gfp;Ccr2-rfp* mice were treated with minocycline (10 mg/kg/day) or saline (as a control) every 2 days, 4 times in total, starting 5 weeks after AAV-BR1-KRAS^G12V^ injection. For testing treatment with mature bAVMs (Figure 7), C57BL/6J mice were treated with minocycline via intraperitoneal injection (10 mg/kg/day) every 2 days, starting from 6 weeks until 10 weeks after AAV-BR1-KRAS^G12V^ injection, for a total duration of 4 weeks.

### Statistics.

Statistical analyses were performed using Prism 10 (GraphPad Software). Statistical significance was determined by unpaired *t* test to compare between 2 groups or ANOVA for multiple groups. All data are presented as mean ± SEM. Significant differences were considered at a *P* value of less than 0.05.

### Study approval.

All procedures involving animal care and experiments were conducted in accordance with the ethical standards described in the NIH *Guide for the Care and Use of Laboratory Animals* (National Academies Press, 2011) and followed the ARRIVE guidelines. All animal experiments were approved by the UTHealth Houston CLAMC (AWC-23-0104, IBC-23-048, CSC-24-012). For human study, studies were performed under the guidance of approved IRB (HSC-MS-20-0053), and with written informed consent was received prior to participation at the Memorial Hermann Hospital.

### Data availability.

Bulk RNA-seq and scRNA-seq data are available in public repositories at dbGAP (accession no. phs002624.v2.p1) and Supplemental Tables 2–6. The supplemental material includes additional methods and Supporting Data Values for all source data in graphs. Detailed protocols or other original data, including deidentified or anonymized human participant data, can be shared upon reasonable request by contacting the corresponding author.

## Author contributions

HP, JEP, BSKY, SMT, EK, and ESP developed experimental methods, performed experiments, and analyzed the datasets. JK provided the plasmid for the bEC-specific (AAV-BR1) vector. HP, JEP, SMT, and AKS performed mouse experiments and maintained mouse lines. SH established the MRI/A protocol and performed the scans. PRC provided human bAVM samples. HP, PRC, EK, and ESP analyzed human bAVM tissue. BSKY and ESP analyzed human bAVM scRNA-seq data. HP, JEP, EK, and ESP quantified imaging data. HAC, JA, PRC, EK, and ESP conceptualized the study. HP, JEP, and ESP designed experiments. JPJS, HAC, SPM, JA, PRC, and EK supported acquiring funding for the project. HP, JEP, BHF, BSKY, JPJS, and ESP wrote/edited the original draft of the manuscript. HP, JEP, BHF, BSKY, SMT, AKS, JPJS, SH, JK, HAC, SPM, JA, PRC, EK, and ESP edited the final manuscript. ESP supervised the study.

## Funding support

This work is the result of NIH funding, in whole or in part, and is subject to the NIH Public Access Policy. Through acceptance of this federal funding, the NIH has been given a right to make the work publicly available in PubMed Central.

- NIH grants R01NS126415 (to ESP) and R01NS135153 (to EK).
- The Aneurysm and AVM foundation (to ESP).
- AVM Research Foundation (to PRC).
- The Dipaolo and Theaker families (to PRC).

## Supplementary Material

Supplemental data

Supplemental table 1

Supplemental table 2

Supplemental table 3

Supplemental table 4

Supplemental table 5

Supplemental table 6

Supplemental table 7

Supplemental video 1

Supplemental video 2

Supplemental video 3

Supporting data values

## Version 1 02/05/2026

In-Press Preview

## Version 2 03/23/2026

Electronic publication
