# Supplementary material for: Endothelial oncogenic KRAS mutation drives the dynamics of microglia and macrophages in brain arteriovenous malformation
Source: JCI Insight. 2026 Feb 5;11(6):e195638. doi: 10.1172/jci.insight.195638 (PMC13043085; doi:10.1172/jci.insight.195638)
Supplement: Supplemental data [file jciinsight-11-195638-s107.pdf]

Supplemental data

Endothelial oncogenic KRAS mutation drives the dynamics of microglia and macrophages in brain arteriovenous malformation

Hyejin Park[1], Jung-Eun Park[1], Bridger H. Freeman[1], Bosco Seong Kyu Yang[1], Shun-Ming Ting[4], Alexander Suh[1], Jude P.J. Savarraj[1], Shuning Huang[2], Jakob Körbelin[3], Huimahn Alex Choi[1], Sean P. Marrelli[4], Jaroslaw Aronowski[4,5], Peng Roc Chen[1]; Eunhee Kim[1], Eun S. Park [1,5]

[1]Vivian L. Smith Department of Neurosurgery, McGovern Medical School, The University of Texas Health Science Center at Houston, Houston, TX 77030, USA.

[2]Department of Diagnostic and Interventional Imaging, McGovern Medical School, The University of Texas Health Science Center at Houston, Houston, TX 77030, USA.

[3]Department of Oncology, Hematology and Bone Marrow Transplantation, University Medical Center Hamburg-Eppendorf, 20246 Hamburg, Germany.

[4]Department of Neurology, McGovern Medical School, The University of Texas Health Science Center at Houston, Houston, TX 77030, USA.

[5]Center for Neuroimmunology and Glial Biology, The Brown Foundation Institute of Molecular Medicine, The University of Texas Health Science Center at Houston, Houston, TX 77030, USA.

Corresponding author: Eun S. Park (E.S.P.), 6431 Fannin Street, Houston, TX, 77030. Phone: +1 (713) 500-5534. Email: Eunsu.park@uth.tmc.edu

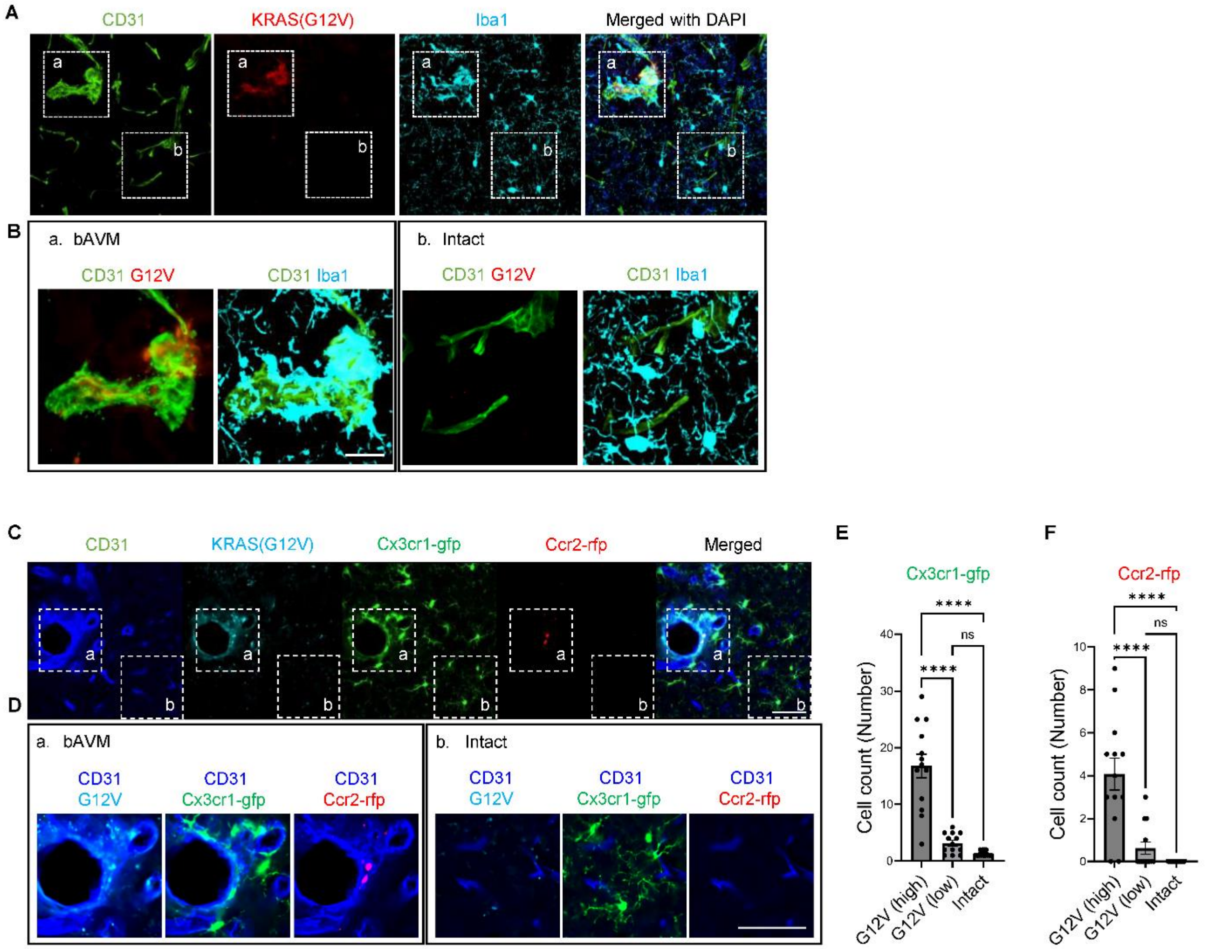

Supplemental Figure 1. AAV-BR1-KRAS^{G12V} drives the expression of KRAS^{G12V} in ECs that are enriched with MG/Mϕ in malformed vessels in KRAS$^{G12V/bEC}$ mice. (**A**) Representative immunofluorescence image demonstrating expression of KRAS^{G12V} in cerebral blood vessels of KRAS$^{G12V/bEC}$ mice at 6 weeks post-AAV-BR1-KRAS^{G12V} injection. (**a**) The dysplastic vessels expressed KRAS^{G12V} and were surrounded by the highly activated Iba1+ MG/Mϕ, which clustered adjacent to malformed vessels expressing KRAS^{G12V}. (**b**) Intact vessels without KRAS^{G12V} expression displayed MG in a resting state. Images are acquired by maximal projection. Z stacks: 30 µm, Scale bar = 50 µm. (**B**) Magnified images of (**a**) bAVM and (**b**) Intact areas. The morphology of MG/Mϕ

appears to depend on $KRAS^{G12V}$ expression on CD31+ vessels. Images are acquired by maximal projection. Z stacks: 30 μm, Scale bar = 20 μm. (**C**) Representative immunofluorescence image demonstrating expression of $KRAS^{G12V}$ in cerebral blood vessels of Cx3cr1-gfp/Ccr2-rfp mice at 6 weeks post-AAV-BR1-$KRAS^{G12V}$ injection. (**D**) Magnified images of (**a**) bAVM and (**b**) Intact areas. (**a**) The dysplastic vessels expressed $KRAS^{G12V}$ and were surrounded by the highly activated Cx3cr1-gfp MG and Ccr2-rfp Mϕ, which clustered adjacent to malformed vessels expressing $KRAS^{G12V}$. (**b**) Intact vessels without $KRAS^{G12V}$ expression displayed Cx3cr1-gfp MG in a resting state. The morphology of MG/Mϕ appears to depend on $KRAS^{G12V}$ expression on CD31+ vessels. Images are acquired by maximal projection. Z stacks: 30 μm, Scale bar = 50 μm. (**E and F**) Bar graphs quantifying cell numbers of total Cx3cr1-gfp+ MG (**E**) or Ccr2-rfp+ Mϕ (**F**) in the intact and bAVM territory, distinguished by $KRAS^{G12V}$ (high) and $KRAS^{G12V}$ (low) area, which were categorized by pixel value lower or higher than 20 (A.U.) ANOVA. ****, $p<0.0001$. Each dot indicates a randomly selected ROI (n=13) obtained from mice (n=5) per group. ns indicates no significant differences.

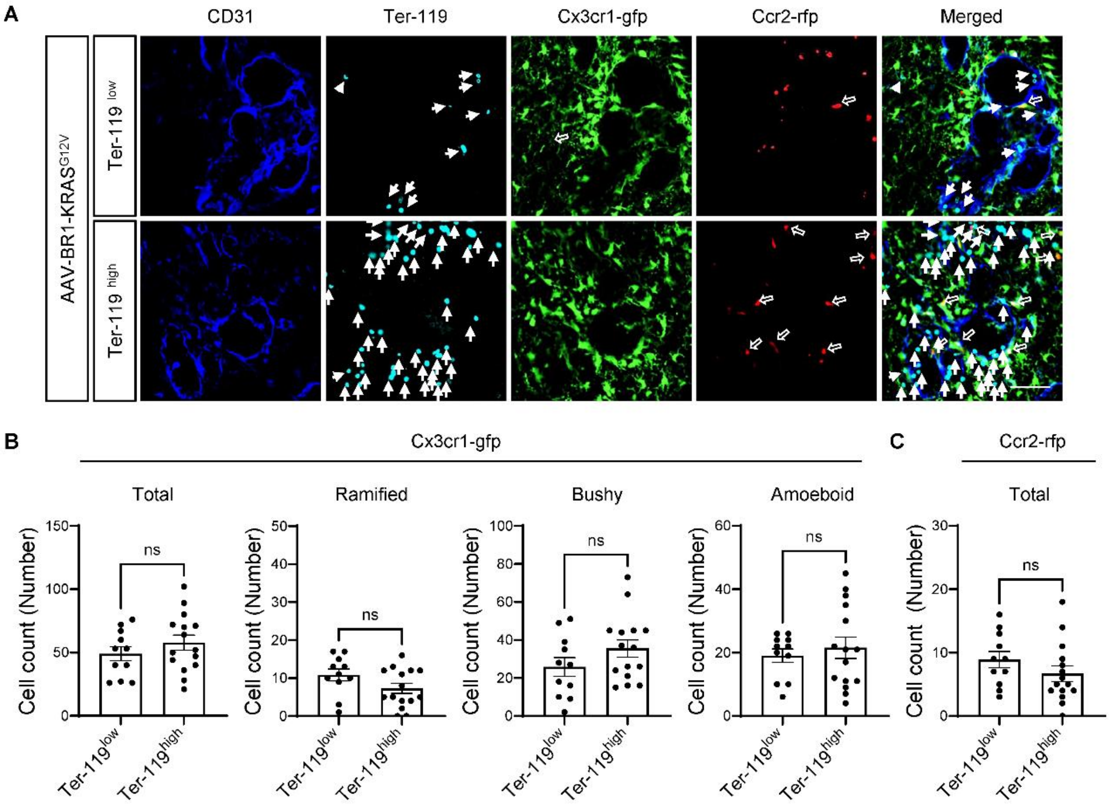

Supplemental Figure 2. Cx3cr1-gfp+ MG and Ccr2-rfp+ Mϕ are co-clustered around RBCs in bAVM territory. (**A**) Representative immunofluorescence image showing the distribution of Cx3cr1-gfp+ MG and Ccr2-rfp+ Mϕ in ruptured bAVM area at 6 weeks post-AAV-BR1-KRAS^{G12V} injection. Note that numerous GFP+/RFP+ cells (open arrows) are detected around highly infiltrated RBCs (Ter-119^{high}) in bAVM territory compared to Ter-119^{low}. Scale bar = 50 μm. Arrows indicate Ter-119+ RBCs. (**B, C**) Bar graphs quantifying the number of Cx3cr1-gfp cells morphologically quantified by ramified, bushy, or amoeboid (**B**), and Ccr2-rfp^{+} cells (**C**), which were classified by Ter-119^{high} and Ter-119^{low} bAVM area. Ter-119^{low} bAVM territories were categorized as having fewer than 15 Ter-119+ RBCs in parenchyma. Unpaired t-test. ns indicates no significant differences. Each dot indicates a randomly selected ROI (n=12-15) obtained from mice (n=5) per group.

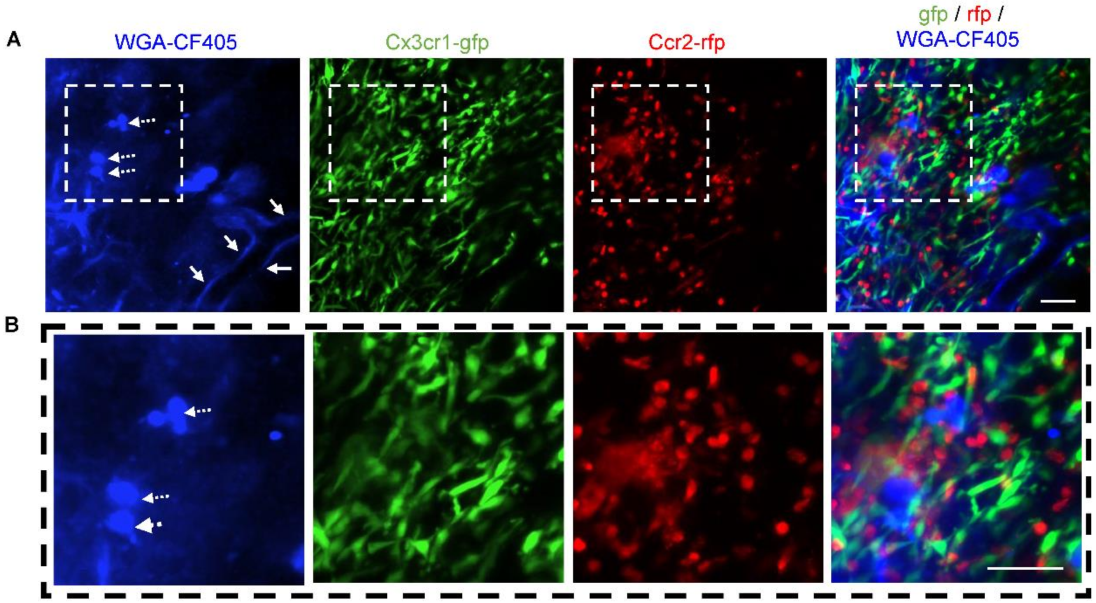

Supplemental Figure 3. Nonlinear WGA-CF405-labeled vessels co-exist with activated microglia and infiltrated macrophages. (**A**) Representative intravital images showing discontinued WGA-CF405-labeled vessels (dotted arrows). Note that WGA-CF405 extravasation highly co-existed with abundant Cx3cr1-gfp+ MG or Ccr2-rfp+ Mϕ. The arrows indicate the WGA-CF405-labeled linear vessels. Images were acquired by maximal projection, z stacks = 32 μm. Scale bar = 50 μm. (**B**) Magnified images of inset. WGA-CF405-labelled vessels display discontinued and tangled vessel structures (dotted arrows). Scale bar = 50 μm.

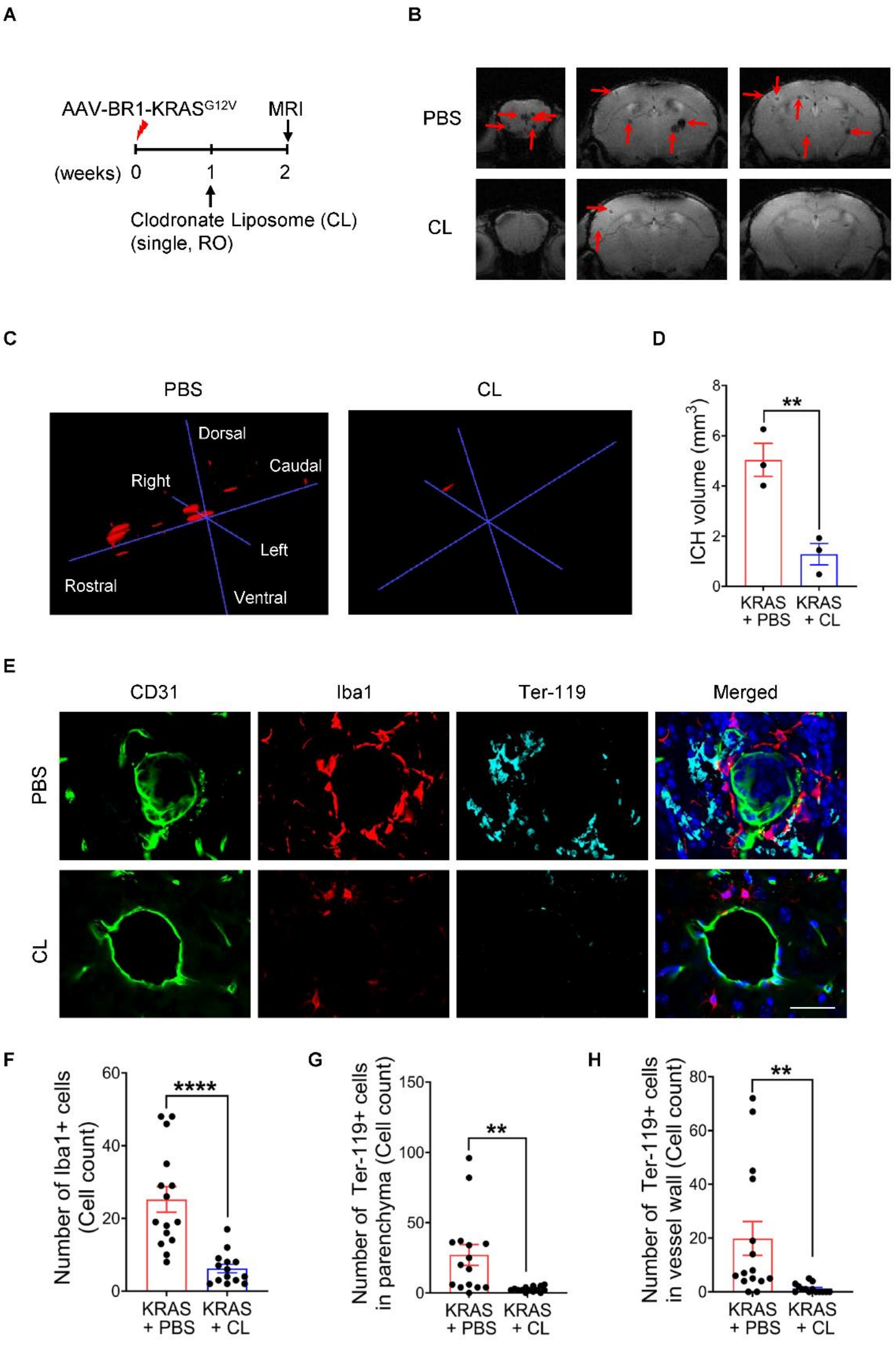

A
B
AAV-BR1-KRAS^{G12V}
MRI
(weeks) 0 1 2
Clodronate Liposome (CL)
(single, RO)
PBS
CL
C
D
PBS
CL
Dorsal
Caudal
Right
Left
Rostral
Ventral
ICH volume (mm^3)
**
KRAS + PBS
KRAS + CL
E
CD31
Iba1
Ter-119
Merged
PBS
CL
F
G
H
Number of Iba1+ cells (Cell count)
****
Number of Ter-119+ cells in parenchyma (Cell count)
**
Number of Ter-119+ cells in vessel wall (Cell count)
**
KRAS + PBS
KRAS + CL

Supplemental Figure 4. MG/Mϕ absence in early stage reduce hemorrhage in KRAS$^{G12V/bEC}$ mice. (**A**) Clodronate liposomes (CL) or PBS (as a control) were administered at 1-week post-AAV-BR1-KRAS^{G12V} injection. Brains were harvested 2 weeks post-AAV-BR1-KRAS^{G12V} injection. (**B**) Representative T_2*-weighted MRI and (**C**) ITK-SNAP volumetric images showing a reduced ICH volume in KRAS$^{G12V/bEC}$ mice. (**D**) Bar graphs quantifying the ICH volume which measured by ITK-SNAP. Unpaired t-tests. **, p<0.001. Each dot indicates an individual mouse (n=3) per group. (**E**) Representative immunofluorescence images showing a reduced Iba1+ MG/Mϕ and Ter119+ RBCs in CL-treated KRAS$^{G12V/bEC}$ mice compared to PBS-treated mice. Scale bar = 50 μm. (**F-H**) Bar graphs quantifying Iba1+ MG (**F**), and Ter-119+ RBC in the parenchyma (**G**) and in blood vessels (**H**). Unpaired t-tests. **, p<0.01, ***, p<0.001. Each dot indicates a randomly selected ROI (n=14-15) obtained from mice (n=4-3) per group.

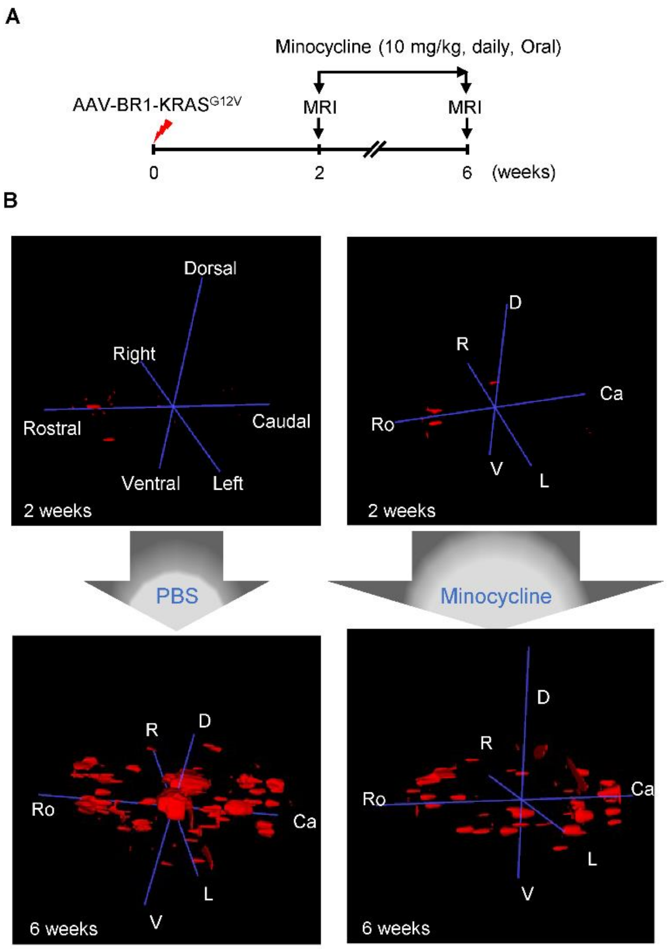

Supplemental Figure 5. Minocycline treatment attenuates ICH occurrence in KRAS$^{G12V/bEC}$ mice. (**A**) The mice received minocycline starting 2 weeks post-AAV-BR1-KRAS^{G12V} injection for 4 weeks. The mice were scanned with T_2*-weighted MRI at 2 and 6 weeks. (**B**) Representative ITK-SNAP volumetric images show that at 6 weeks, minocycline attenuated the occurrence of ICH in minocycline-treated KRAS$^{G12V/bEC}$ mice compared to PBS-treated KRAS$^{G12V/bEC}$ mice, despite similar results between groups at the 2-week, pre-treatment scan.

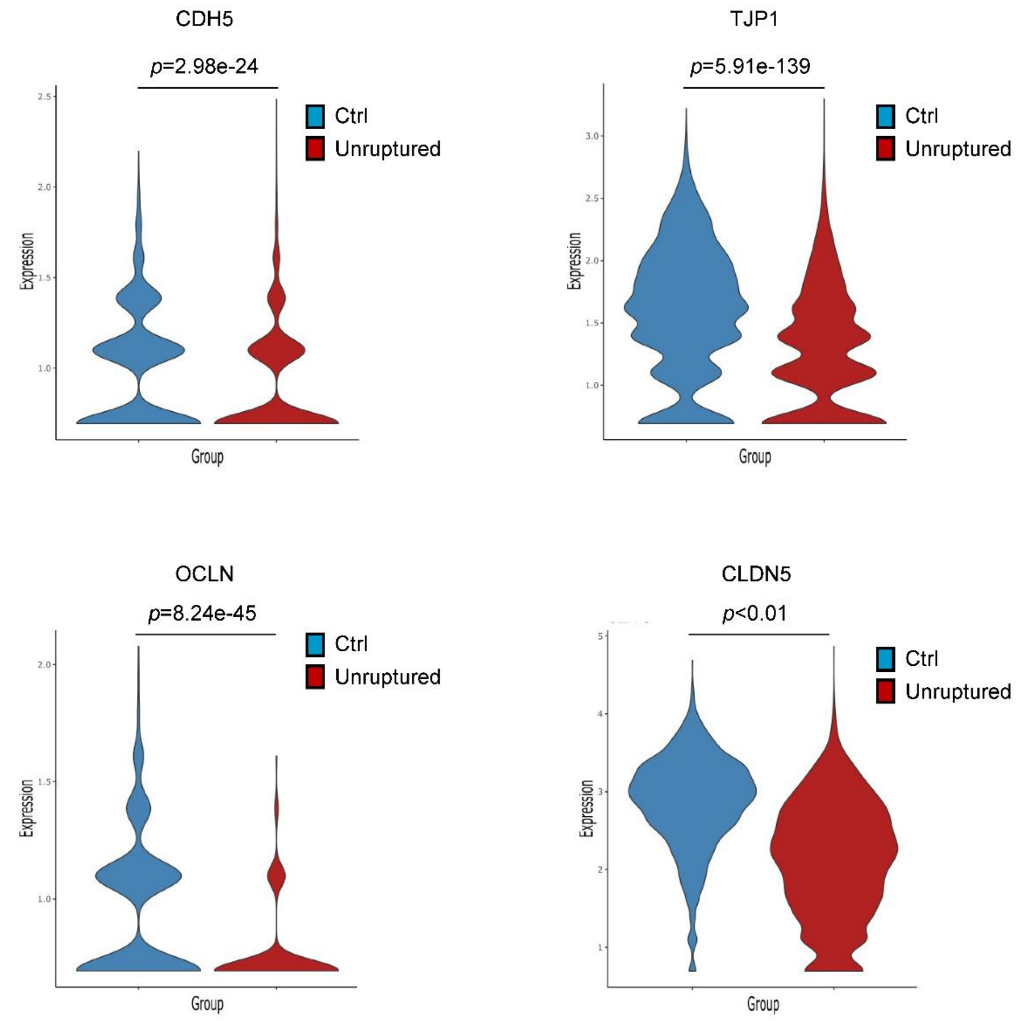

Supplemental Figure 6. Loss of BBB-comprising genes in human unruptured bAVM patients. *External validation:* Violin plot visualizes the decreased CDH5, TJP1, OCLN, and CLDN5 expression in ECs from unruptured bAVM tissues compared to control samples that had not been previously analyzed in the Winkler et al., 2022 Science. The black dots indicate the gene values for each cell. The significance of selected genes was tested with Wilcoxon rank-sum tests to compare Ctrl (control) vs. unruptured. Log2 fold changes and *p*-values were indicated on violin plots. ns indicates no significant differences.

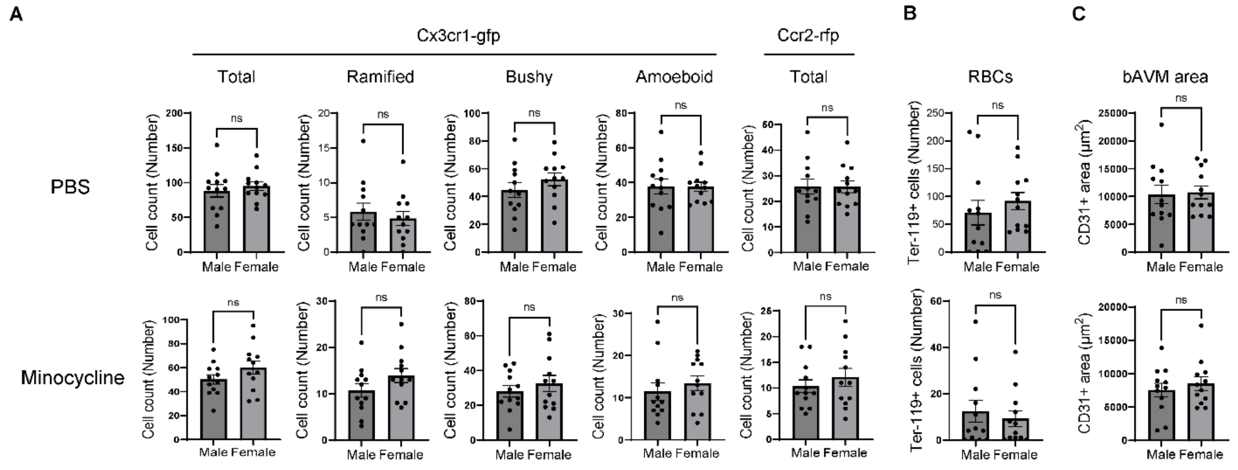

Supplemental Figure 7. Minocycline treatment does not exhibit sex differences in MG/Mϕ activation/infiltration, hemorrhagic conversion, and bAVM area in bAVM mice. Six-week-old male and female Cx3cr1-gfp;Ccr2-rfp mice were injected with AAV-BR1-KRAS^{G12V}, followed by treatment with minocycline (10 mg/kg, daily, oral) or PBS starting 2 weeks post-AAV-BR1-KRAS^{G12V} injection for 4 weeks. The mice were allowed a drug-off period from 6 to 10 weeks post-AAV-BR1-KRAS^{G12V} injection, and were sacrificed for analysis of sex differences. (**A-C**) Bar graphs quantifying the number of Cx3cr1-gfp MG and Ccr2-rfp Mϕ, which were morphologically classified as ramified, bushy, and amoeboid, and total GFP and RFP cells (**A**), infiltrated Ter-119+ RBCs (**B**), and CD31+ bAVM area (**C**) in PBS- or minocycline-treated male and female bAVM mice. Unpaired t-test. ns indicates no significant differences. Each dot indicates a randomly selected ROI (n=12) from mice (n=6) per group.

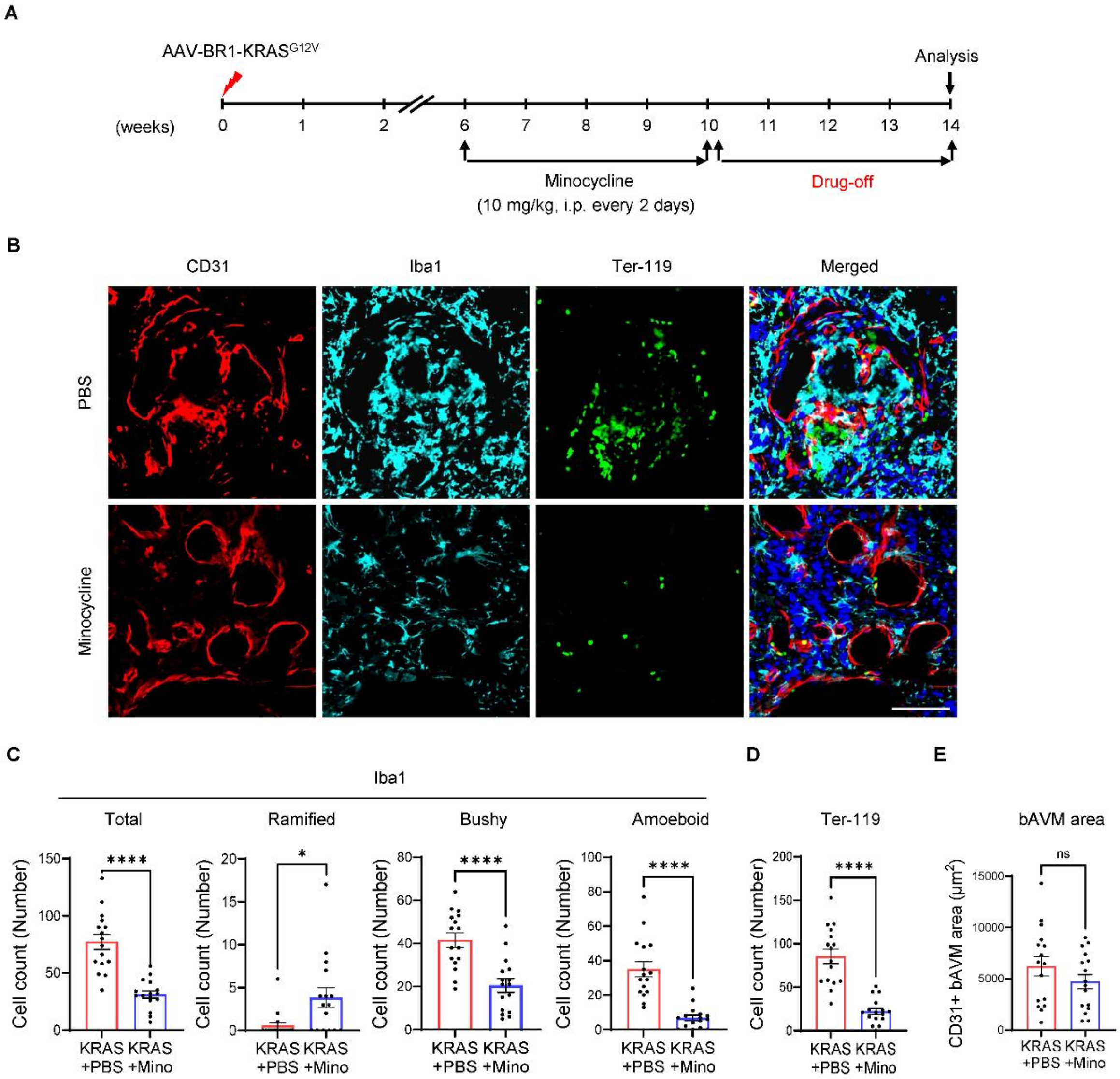
A
AAV-BR1-KRAS^{G12V}
Analysis
(weeks) 0 1 2 6 7 8 9 10 11 12 13 14
Minocycline
(10 mg/kg, i.p. every 2 days)
Drug-off
B
CD31
Iba1
Ter-119
Merged
PBS
Minocycline
C
Iba1
Total
Ramified
Bushy
Amoeboid
Cell count (Number)
****
*
KRAS +PBS
KRAS +Mino
D
Ter-119
E
bAVM area
ns
CD31+ bAVM area (μm^2)

Supplemental Figure 8. Post-treatment with minocycline exerts a sustained inhibitory effect on MG/Mϕ activation/infiltration in bAVM following treatment cessation. (**A**) bAVM mice received minocycline starting 6 weeks post-AAV-BR1-KRAS^{G12V} injection, followed by drug withdrawal for 4 weeks. (**B**) Representative immunofluorescence images showing attenuated cluster of Iba1+ MG/Mϕ, and Ter-119+ RBCs around CD31+ (vessel, cyan) bAVM territory in KRAS$^{G12V/bEC}$ mice treated with minocycline compared to PBS. Scale bar: 50 μm. (**C-E**) Bar graphs quantifying cell numbers of Iba1+ MG/Mϕ for total, ramified, bushy, and amoeboid based on morphology assessment (**C**), numbers of infiltrated Ter-119+ RBCs in parenchyma (**D**), or area of CD31+ bAVM (**E**) in the bAVM territory between bAVM mice treated with minocycline or PBS. Unpaired t-test. *, $p<0.05$; ****, $p<0.0001$. ns indicates no significant differences. Each dot indicates a randomly selected ROI (n=16) from mice (n=5) per group.

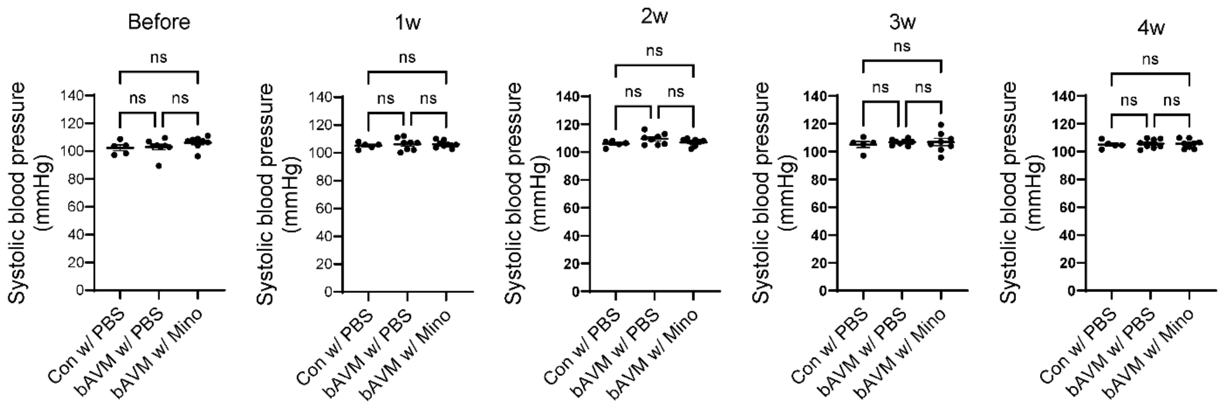

Supplemental Figure 9. Minocycline does not change systolic blood pressure in KRAS$^{G12V/bEC}$ mice. The 6-week-old C57BL/6 mice were injected with AAV-BR1-KRAS^{G12V} and treated with minocycline (10 mg/kg, i.p., every 2 days) starting 6 weeks post-AAV injection for 4 weeks. Systemic systolic blood pressure was longitudinally monitored in bAVM mice treated with minocycline (bAVM w/ Mino) compared to PBS (bAVM w/ PBS) or control mice treated with PBS (Con w/ PBS) at baseline (Before) and weekly up to 4 weeks. One-way ANOVA. ns indicates no significant differences. Each dot indicates an individual mouse per group (n=5-8).

Supplemental Table 1. Human bAVM tissues and controls were used for Figure 2. Human brain AVM tissues and control superficial temporal artery (STA, control) were dissected from bAVM patients from the local operating room. Studies were performed with written informed consent at the Memorial Hermann Hospital. A small segment of the STA does not alter the blood supply.

Supplemental Table 2. Systolic blood pressure during treatment periods between KRAS$^{G12V/bEC}$ mice and control mice. C57BL/6 mice received intraperitoneal injections of either PBS or minocycline (10 mg/kg/day, i.p) every two days, starting 6 weeks post-AAV-BR1-KRAS^{G12V} injection, for 4 weeks. Blood pressure was measured weekly before starting minocycline treatment and throughout the treatment period. Data are presented as mean ± SD.

Supplemental Table 3. Differential immune cell proportion between ruptured and unruptured bAVM (Re-analyzed bulk RNA-seq from Winkler et al., 2022 Science).

Supplemental Table 4. Expression level of AIF1 in MG between control and unruptured bAVM (Re-analyzed scRNA-seq from Winkler et al., 2022 Science).

Supplemental Table 5. Expression level of CD68 in MG between control and unruptured bAVM (Re-analyzed scRNA-seq from Winkler et al., 2022 Science).

Supplemental Table 6. Expression level of AIF1 in Mϕ between control and unruptured bAVM (Re-analyzed scRNA-seq from Winkler et al., 2022 Science).

Supplemental Table 7. Expression level of CD68 in Mϕ between control and unruptured bAVM (Re-analyzed scRNA-seq from Winkler et al., 2022 Science).

Supplemental Video 1. Three-dimensional rendering of the bAVM from the KRAS$^{G12V/bEC}$ mouse. The movie shows CD31+ (green) vessels and Iba1+ MG/Mϕ (red) surrounding thick and dysplastic bAVM, corresponding to Figure 1A. 509.12 x 509.12 x 66 μm.

Supplemental Video 2. Three-dimensional rendering of the human bAVM nidus. The movie depicts dense Iba1+ MG/Mϕ (Red) surrounding thick and dysplastic CD31+ (Green) vessels, as shown in Figure 2A. 318.20 x 318.20 x 134 μm.

Supplemental Video 3. Intravital time-lapse of the bAVM from the KRAS$^{G12V/bEC}$ mouse. The movie presents Ccr2-rfp+ Mϕ (red) infiltrating from the lumen into the parenchyma across dysplastic vessels (Blue) in Cx3cr1-gfp;Ccr2-rfp mice-injected with AAV-BR1-KRAS^{G12V}, corresponding to Figure 3H. 114.35 x 114.35 μm.

Supplemental Methods

Endothelial oncogenic KRAS mutation drives the dynamics of microglia and macrophages in brain arteriovenous malformation

Hyejin Park[1], Jung-Eun Park[1], Bridger H. Freeman[1], Bosco Seong Kyu Yang[1], Shun-Ming Ting[4], Alexander Suh[1], Jude P.J. Savarraj[1], Shuning Huang[2], Jakob Körbelin[3], Huimahn Alex Choi[1], Sean P. Marrelli[4], Jaroslaw Aronowski[4,5], Peng Roc Chen[1]; Eunhee Kim[1], Eun S. Park [1,5]

[1]Vivian L. Smith Department of Neurosurgery, McGovern Medical School, The University of Texas Health Science Center at Houston, Houston, TX 77030, USA.

[2]Department of Diagnostic and Interventional Imaging, McGovern Medical School, The University of Texas Health Science Center at Houston, Houston, TX 77030, USA.

[3]Department of Oncology, Hematology and Bone Marrow Transplantation, University Medical Center Hamburg-Eppendorf, 20246 Hamburg, Germany.

[4]Department of Neurology, McGovern Medical School, The University of Texas Health Science Center at Houston, Houston, TX 77030, USA.

[5]Center for Neuroimmunology and Glial Biology, The Brown Foundation Institute of Molecular Medicine, The University of Texas Health Science Center at Houston, Houston, TX 77030, USA.

Corresponding author: Eun S. Park (E.S.P.), 6431 Fannin Street, Houston, TX, 77030. Phone: +1 (713) 500-5534. Email: Eunsu.park@uth.tmc.edu

Genotyping for Animals

The standard PCR protocols were performed with the following primers. *Cx3cr1-gfp;Ccr2-rfp* dual-reporter mice: Cx3cr1, Wildtype Forward (5'GTCTTCACGTTCGGTCTGGT), Common (5'CCCAGACACTCGTTGTCCTT), Mutant Forward (5'CTCCCCCTGAACCTGAAAC); Ccr2, Common (5'TAAACCTGGTCACCACATGC), Wildtype Reverse (5'GGAGTAGAGTGGAGGCAGGA), Mutant Reverse (5'CTTGATGACGTCCTCGGAG). *Cx3cr1-cre/ERT2;Rosa26-iDTR* mice: Cx3cr1-cre/ERT2 primer, Wildtype forward (5'AGCTCACGACTGCCTTCTTC), mutant forward (5' GTTAATGACCTG CAGCCAAG), Common (5'ACGCCCAGACTAATGGTGAC); and Rosa26-iDTR primer, Common (5' AAAGTCGCTCTGAGTTGTTAT), Mutant (5'GCGAAGAGTTTGTCCTCAACC), Wildtype reverse (5'GGAGCGGGAGAAATGGATATG).

Cranial window installation and Intravital imaging

Cx3cr1-gfp;Ccr2-rfp mice were anesthetized by inhalation with isoflurane (3%; induction of anesthesia 1.5%; maintenance during surgical procedure) (NDC66794-017-25, Piramal Pharma Limited, Kohir mandal, Telangana, India). Animals were fixed in a stereotaxic frame (Stoleting, USA) for full craniotomy, and the body temperature was kept warm using a heating pad (RH-0806C, Onkey Electronic Technology, China). After removal of hair, the skin over each animal's head was cleaned with 70% alcohol and disinfected with povidone-idodine (Betadine, 67618-150-17, Avrio Health, Stanford, CA, USA) three times before being cut with surgical scissors. Then, the epidermal skin of the skull was gently resected using a cotton tip. A hole (4 mm diameter) on the skull overlying

the cortex of the right hemisphere was made using dental drill (HP4-310, Foredom Electric Co., Bethel, CT, USA). For the glass top window, a glass coverslip (5 mm diameter) (72296-05, Electron Microscopy Sciences, Hatfield, PA, USA) was fashioned over the hole in the skull. Edges of the window were sealed with dental resin (Fusion Flo) (20009, Prevest DenPro, Digiana, Jammu, India). The applied dental resin was then hardened by exposure to blue LED light (180015, Denshine, China). Meloxicam (1 mg/kg) was administered via intraperitoneal injection for 3 days following the procedure to reduce pain. The mice are allowed spontaneous recovery time for 4 weeks after the procedure. At 4 weeks post-cranial window installation, animals of each group were again anesthetized under Isoflurane (NDC66794-017-25, McKesson, Irving, TX, USA). Animals were laid on a bed made of paper towel and kept warm with a heating pad. 10% BSA-647 (A34785, Invitrogen, Waltham, MA, USA) or WGA-CF405 (29028, Biotium, Fremont, CA, USA) (0.5 mg/ml, 200μl) was injected into the RO sinus vein to image blood vessels. The head of animal was fixed with head holder (SGM-4, Narishige International USA, INC, Amityville, NY, USA) and was placed under upright confocal microscopy with x25 lens (Nikon A1R-MP, Nikon, Tokyo, Japan) for fluorescence image acquisition.

BBB permeability assay

Six-week-old C57BL/6 mice received a retro-orbital (RO) injection of AAV-BR1-KRAS^{G12V}, followed by a BBB permeability assay at 4 weeks post-AAV injection. Mice were anesthetized with isoflurane, and Alexa Fluor 647-conjugated bovine serum albumin (BSA-647, A34785, Invitrogen, 16 mg/kg) with Linger's solution was administered via RO injection. After 15 minutes, the bAVM mice were perfused with cold PBS, then fixed in 10%

formalin. Coronal brain sections (30 μm) were used for immunofluorescence staining with antibodies of Ter-119, CD31, or Iba1. Images were captured by Confocal microscopy.

Magnetic Resonance Imaging and Magnetic Resonance Angiography

To observe the occurrence of ICH and malformed vasculature, Magnetic Resonance Imaging (MRI) and Magnetic Resonance Angiography (MRA) were performed at the UTHealth preclinical imaging core facility, as previously described (1). Briefly, 1.5% isoflurane in a gas mixture of 30% oxygen and 70% medical air was used for anesthesia. The respiration and body temperature of the mice were maintained with a physiological monitoring system (Small Animal Instruments, Stony Brook, NY, USA) at 80-120 breaths/min and $36.5 \pm 0.5\ ^{o}C$, respectively. A 7 T Bruker BioSpec system (Bruker Biospin, Billerica, MA, USA) equipped with a B-GA12 gradient set was used for all MRI acquisition. ImageJ software (National Institutes of Health [NIH]) was used to visualized T_2*-weighted and MRA images. The volume of ICH as revealed by the T_2*-weighted images was measured using ITK-SNAP 4.0.2 (GNU General Public License). ICH lesions in the $KRAS^{G12V/bEC}$ mice were further validated through the comparison with mice injected with AAV-BR1-eGFP mice to distinguish the ICH lesion and anatomical negative regions. The measurement of ICH volume was performed by a blinded researcher.

Immunohistochemistry

Human bAVM and STA were washed with cold phosphate-buffered saline (PBS) and fixed with 10% formalin for 5 hours at 4 ^{o}C. After 24 hours, the brain was immersed in 30% sucrose and 0.1% sodium azide solution for 24 hours. The brain was embedded with

optimal cutting temperature compound (OCT, 23-730-571, Fisher scientific, Hampton, NH, USA). The human bAVM tissues and STA were used for frozen sectioning by 10 μm thickness by cryostat (CM1520, Leika Biosystems, Germany). The brains from $KRAS^{G12V/bEC}$ mice were coronally sectioned at 30 μm. All sections were incubated with antigen retrieval buffer (citrated buffer, pH 6.0) at 80 ^{o}C for 20 min before staining with primary antibodies. The sections were washed in cold phosphate-buffered saline (PBS) for 10 min and block with blocking solution (1% BSA, 0.3% Triton X-100, 0.02% Tween 20, 10% donkey serum in PBS) for 1 h at room temperature. Primary antibodies of sheep anti-CD31 (1: 200, AF806, R&D, Minneapolis, MN, USA), Goat anti-CD31 (1:200, AF3628, R&D system, Minneapolis, MN, USA), Rabbit anti-KRAS (G12V mutant) (1: 200, MA5-42375, Invitrogen, Waltham, MA, USA), Rabbit anti-Iba1 (019-19741, Wako, Chuo-ku, Osaka Japan), Mouse anti-IL-1β (1:500, 12242, Cell Signaling Technology, Danvers, MA), Rat anti-IL-6 (1:500, 14-7061-85, Invitrogen, Waltham, MA, USA), Rat anti-Ter-119 (1:200, MAB1125, R&D system, Minneapolis, MN, USA), and Rabbit anti-VE-Cadherin (1:200, 36-1900, Invitrogen) were diluted in 1 % BSA and 0.3% Triton X-100 in PBS. For the observation of primary antibodies under the fluorescent microscopy, secondary antibodies of Alexa 488 conjugated anti-mouse or goat (1:500, 715-545-150, 705-545-147), Rhodamine Red-conjugated-anti rabbit (1:500, 711-295-152), and Alexa 647-conjugated-anti rabbit (1:500, 711-605-152), all from Jackson Immunoresearch (West Grove, PA, USA), and Alexa 405-conjugated- anti goat (1:200, A-31553) from Invitrogen were diluted in 1 % BSA and 0.3% Triton X-100 in PBS. The stained sections were mounted using 4',6-diamidino-2-phenylindole (DAPI)-Fluoromount-G Clear mounting media (OB010020, SouthernBiotech, Birmingham, AL, USA). The stained sections were

observed using confocal microscopy (Nikon A1R, Nikon, Japan, Leica DM4000 B LED) and quantified using ImageJ software (NIH).

To quantify the number of Cx3cr1-gfp- and Ccr2-rfp-expressing MG/Mϕ around intact blood vessels and bAVM areas, we performed the quantification within images acquired with the digital 37.5X zoom of a confocal microscope (Nikon). To quantify the gfp-MG, the MG morphology is analyzed by ImageJ-Sholl analysis (NIH), in accordance with our previously presented representative images of ramified, bushy, and amoeboid form of MG (1, 2). Briefly, the maximum radius of the cell soma and the radius surpassing the longest branch of the cells were automatically measured by the program as shown in the image below, and we categorized i) ramified, cell soma (0-6 of maximum radius 27), branch (0-27); ii) activated/bushy, cell soma (0-10), branch (0-27); and iii) amoeboid, cell soma (0-3), branch (0-5). The quantification for the number of each category of Iba1+ MG was performed using randomly selected ROIs for bAVMs and comparable intact areas.

bAVM Tissue clearing and immunostaining

Formalin-fixed human bAVM tissues and brains harvested from $KRAS^{G12V/bEC}$ mice were dehydrated with 30% sucrose solution. The brains were sectioned with 2 mm thickness according to mouse brain matrix (RBMA200C, World Precision Instrument, Sarasota, FL, USA). The brain clearing process for was performed with Binaree tissue clearing rapid system (BRTC, Binaree, Daegu, South Korea) with the Rapid clearing solution for 6 hours. The cleared tissue was permeabilized at 37 ^{o}C and incubated for 3-5 days with the following primary antibodies: Goat Anti-CD31 (1:200, AF3628, R&D system, Minneapolis, MN, USA), sheep anti-CD31 (1: 200, AF806, R&D system, Minneapolis, MN, USA), or

Rabbit anti-Iba1 (019-19741, Wako, Chuo-ku, Osaka Japan). Then, the cleared tissue was incubated for 3-5 days with the following secondary antibodies: Alexa 488 conjugated anti-mouse or goat (1:400, 705-545-147,715-545-150), Alexa 647-conjugated-anti rabbit (1:400, 711-605-1520 all from Jackson Immunoresearch (West Grove, PA, USA)) with PBS. The fluorescent images were captured by upright confocal microscopy with x25 lens (Nikon A1R, Nikon, Japan).

Image analysis

All images were acquired under the same conditions in each experiment by confocal microscopy. All Cx3cr1-gfp- or Ccr2-rfp- cells were quantified on corresponding ROIs of the bAVM territories, which were identified by CD31+ enlarged vessel morphology. Cx3cr1-gfp-/Ccr2-rfp-expressing cells and Ter119+ erythrocytes on immunostained images were quantified using a cell counter from ImageJ within the same ROIs (339.41 μm x 339.41 μm). To quantify Cx3cr1-gfp- or Ccr2-rfp-expressing cells on images acquired from intravital imaging, all images are processed within the same range of fluorescence intensity. Individual areas containing Cx3cr1-gfp-expressing cells were designated by auto local threshold (ImageJ). The relative cell area (μm^2) was measured by particle analysis from ImageJ. The Ccr2-rfp cells were counted using cell counter from ImageJ with the same ROIs (367.85 μm x 367.85 μm). To quantify the fluorescent intensity of VE-cadherin, the mean intensity of VE-cadherin+ was calculated by masking the endothelial area only. Image processes were performed by Image J, and calculations were performed using MATLAB with the same ROI of confocal images (339.41 μm x 339.41 μm).

Real-time quantitative PCR

The resected brain tissues from KRAS$^{G12V/bEC}$ mice and AAV-BR1-eGFP-injected mice (as a control) were used to extract total mRNA using a Trizol reagent (15596026, Invitrogen, Waltham, MA) according to manufacturer's protocols. cDNA was synthesized using a commercial kit (High-Capacity cDNA Reverse Transcription Kits, 4368814, Applied Biosystems, Foster City, CA) and the mRNA expression levels were measured by Real-Time *qRT-PCR* reaction (Applied Biosystems Quant 3 Studio qPCR) using amfisure qGreen master mix (Q5603-005, GenDEPOT). PCR primers were purchased from Sigma-Aldrich (St. Louis, MO). For measuring mRNA levels, the following primers were used: IL6, F-5'TGGTACTCCAGAAGACCAGAGG, R-5'AACGATGATGCACTTGCAGA; MMP2, F-5'CCGATCTACACCTACACCAAGAAC, R-5'CCAGTACCAGTGTCAGTATCAG; TMEM119, F-5'GTGTCTAACAGGCCCCAGAA, R-5'AGCCAGCTGGTATCAAGGAG; CSF1R, F-5'TGAGCAAGACCTGGACAAGGA, R-5'CCGCTGGTCAACAGCACGTTT. Glyceraldehyde 3-phosphate dehydrogenase (GAPDH) was used as an internal control. F-5′GGAGTCAACGGATTTGGTCG, R-5′GGAATCATATTGGAACATGTAAACC. The PCR data were analyzed by comparative CT method (ΔΔCT method) (Applied Biosystems Quant 3 Studio qPCR).

Blood pressure measurement

C57BL/6 mice received intraperitoneal injections of either PBS or minocycline (10 mg/kg/day) every two days, beginning six weeks after AAV-BR1-KRAS^{G12V} injection and continuing until ten weeks post-AAV injection. Systolic blood pressure was measured weekly during the treatment period in conscious mice using the CODA® High Throughput

Non-Invasive Blood Pressure System (Kent Scientific, Torrington, CT, USA), which uses a volume-pressure recording (VPR) sensor and an occlusion tail-cuff method. Mice were placed in individual restrainers on a warming platform maintained at 32-34 °C to ensure optimal tail blood flow. The occlusion and VPR cuffs were positioned near the base of the tail. Each measurement session began with five acclimation cycles, followed by 15 measurement cycles. Blood pressure data were automatically recorded by the CODA® software. For each mouse, five valid measurements were averaged to determine the final systolic blood pressure value.

References

1. Park ES, Kim S, Huang S, Yoo JY, Korbelin J, Lee TJ, et al. Selective Endothelial Hyperactivation of Oncogenic KRAS Induces Brain Arteriovenous Malformations in Mice. *Ann Neurol.* 2021;89(5):926-41.
2. Heindl S, Gesierich B, Benakis C, Llovera G, Duering M, and Liesz A. Automated Morphological Analysis of Microglia After Stroke. *Front Cell Neurosci.* 2018;12:106.
